# Palladin isoforms 3 and 4 regulate cancer-associated fibroblast pro-tumor functions in pancreatic ductal adenocarcinoma

**DOI:** 10.1038/s41598-021-82937-3

**Published:** 2021-02-15

**Authors:** J. I. Alexander, D. B. Vendramini-Costa, R. Francescone, T. Luong, J. Franco-Barraza, N. Shah, J. C. Gardiner, E. Nicolas, K. S. Raghavan, E. Cukierman

**Affiliations:** 1 Cancer Biology and the Marvin and Concetta Greenberg Pancreatic Cancer Institute, Fox Chase Cancer Center, Philadelphia, PA USA; 2 Molecular, Cellular Biology and Genetics Program, College of Medicine, Drexel University, Philadelphia, PA USA

**Keywords:** Cancer, Cell biology

## Abstract

Pancreatic Ductal Adenocarcinoma (PDAC) has a five-year survival under 10%. Treatment is compromised due to a fibrotic-like stromal remodeling process, known as desmoplasia, which limits therapeutic perfusion, supports tumor progression, and establishes an immunosuppressive microenvironment. These processes are driven by cancer-associated fibroblasts (CAFs), functionally activated through transforming growth factor beta1 (TGFβ1). CAFs produce a topographically aligned extracellular matrix (ECM) that correlates with reduced overall survival. Paradoxically, ablation of CAF populations results in a more aggressive disease, suggesting CAFs can also restrain PDAC progression. Thus, unraveling the mechanism(s) underlying CAF functions could lead to therapies that reinstate the tumor-suppressive features of the pancreatic stroma. CAF activation involves the f-actin organizing protein palladin. CAFs express two palladin isoforms (iso3 and iso4) which are up-regulated in response to TGFβ1. However, the roles of iso3 and iso4 in CAF functions remain elusive. Using a CAF-derived ECM model, we uncovered that iso3/iso4 are required to sustain TGFβ1-dependent CAF activation, secrete immunosuppressive cytokines, and produce a pro-tumoral ECM. Findings demonstrate a novel role for CAF palladin and suggest that iso3/iso4 regulate both redundant and specific tumor-supportive desmoplastic functions. This study highlights the therapeutic potential of targeting CAFs to restore fibroblastic anti-tumor activity in the pancreatic microenvironment.

## Introduction

Pancreatic ductal adenocarcinoma (PDAC) will soon be the 2nd leading cause of cancer related deaths in the U.S^1^. A major hallmark of PDAC is a fibrotic-like stromal reaction that constitutes the bulk of the tumor mass, known as desmoplasia. This reaction is produced by fibroblastic cells known as cancer-associated fibroblasts (CAFs) that reside in the vicinity of PDAC cells and produce an extracellular matrix (ECM) that is predictive of patient outcome^2^. Desmoplasia is known to support tumor progression by promoting immunosuppression and limiting nutrients as well as therapeutic perfusion^3,4^. Paradoxically, drugs that inhibit CAFs in the PDAC stroma, or genetic ablation of selective CAF populations, can lead to a more aggressive malignancy^5–7^. This suggests that CAFs, in the desmoplastic stroma, are both pro- and anti-tumorigenic. Thus, delineating the mechanisms by which CAFs influence PDAC cells, within the desmoplastic stroma, may aid in the development of future therapeutic strategies.

CAFs are functionally diverse, yet dynamically interchangeable^8,9^, and can be reciprocally regulated by other cells in the microenvironment as well as by extracellular components and secreted materials accessible at local and distal niches^4^. The activation of fibroblasts into CAFs with a myofibroblastic phenotype is heavily driven by TGFβ1 and leads to the development of many pro-tumor desmoplastic features seen in PDAC. Myofibroblastic CAFs acquire the expression of alpha smooth muscle actin (αSMA), adopt a spindle-like morphology, and produce topographically aligned desmoplastic ECMs (d-ECMs). These characteristics can play both anti and pro-tumoral roles^4,10^. In addition to myofibroblastic functions, CAFs also secrete several types of cytokines, such as IL-6 and IL-8, that help establish an immunosuppressive microenvironment^3,4,11–13^.

The production and remodeling of cancer-associated d-ECM is facilitated by cytoskeletal rearrangements in CAFs, which are mediated in part by the filamentous actin (f-actin) organizing protein palladin^14–16^. Palladin is a scaffolding protein^16–18^ that was identified as an independent prognostic marker in PDAC and potentially predicts survival following chemoradiation^15^. Further, palladin is upregulated in early precancerous lesions and its expression increases during malignant PDAC transformation^19^. Importantly, palladin overexpression is robust in PDAC stroma, and is a conserved trait of activated CAFs in desmoplasia^19,20^. Hence, in this study we posited that stromal palladin function may be important in CAF activation and contribute to the pro-tumorigenic functions of CAFs.

Although there are 9 identified palladin isoforms, only two (iso3 and iso4) have been reported to be expressed in myofibroblasts, in response to TGFβ1^21^. These isoforms serve as major scaffolds for actin bundling proteins, such as αSMA, and are important for cell adhesion, myofibroblastic contraction, and cell motility^18,19^. However, the role of these palladin isoforms in TGFβ1 mediated CAF functions remains unclear. In this study, we explored the roles of iso3 and iso4 in PDAC CAF phenotypes and functions, such as myofibroblastic CAF-mediated d-ECM remodeling, inflammatory cytokine secretions, and d-ECM supported PDAC cell survival. We found that palladin loss phenocopies TGFβ1 inhibition and demonstrated that both palladin isoforms are important for CAFs to produce d-ECMs with tumor supportive functions. Moreover, results seem to suggest an added role for iso4 in further promoting the inflammatory function of CAFs. Taken together, findings from this study suggest that palladin is a key regulator of pro-tumor CAF functions and d-ECM remodeling of the PDAC microenvironment.

## Results

### Palladin isoforms 3 and 4 are upregulated in human PDAC stroma

**Figure 1:**
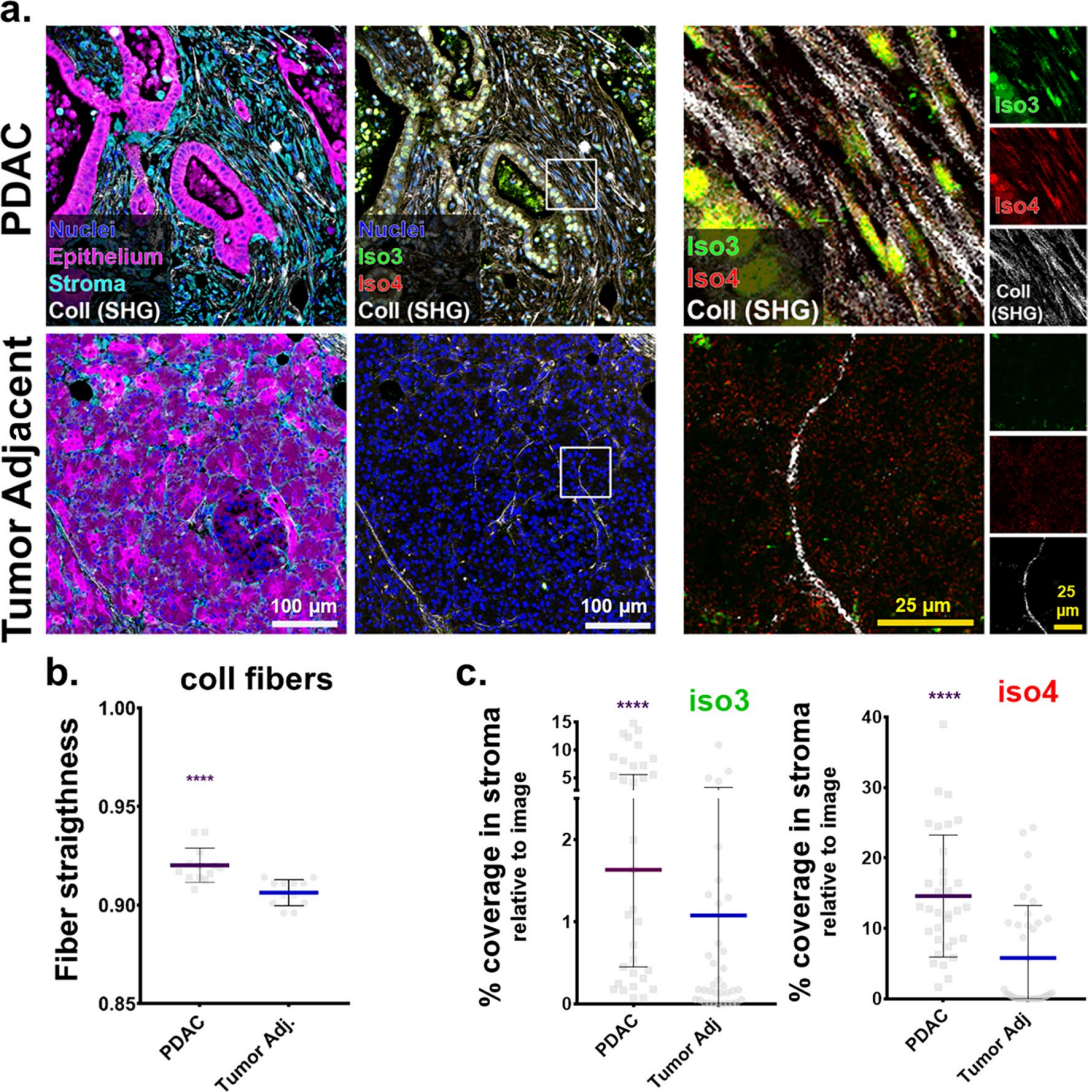
Palladin iso3 & iso4 are upregulated in the fibrillar collagen-rich stroma of human PDAC. (**a**) Representative images depicting epithelial/tumoral areas (magenta) as well as stroma (cyan) areas in surgical tissue sections, pathologically defined as pancreatic cancer (PDAC) or matched normal/benign pancreas (Tumor Adjacent). The images are representative of 3 patient samples that included SMI as well as SHG assessments, while SMI alone was also conducted in 2 additional surgical sample pairs. Epithelial compartments were labeled with antibodies against pan-cytokeratin, EpCAM, and CD-70 identifying all epithelial cells including cancer cells (magenta). Stromal cell compartments were identified using anti-vimentin antibodies (cyan). Nuclei were stained using DRAQ5 dye (blue). Palladin isoforms were identified using antibodies against iso3 (clone G2; green) and iso4 (clone IE6; red). Fibrillar collagen signatures, detected via SHG, are shown in white. Yellow squares correspond to magnified regions, shown to their right, while far right column shows single colored images corresponding to the same magnified regions. Scale bars are provided for each magnification. (**b**) Evaluation of SHG generated signatures assessing straightness of collagen fibers. Values were obtained using CT-FIRE software (see “Material and methods”). Each bullet point represents a single image. Three images were acquired from each of the three paired patient samples for a total of nine images from PDAC, and nine from matching for Tumor Adjacent samples. (**c**) Quantification of the percentage coverage of iso3 or iso4 immunostaining within vimentin positive areas, relative to the total image area. Analysis was carried out using readouts generated by the SMIA-CUKIE 2.1.0 software (see “Materials and methods”). Data presented in (**c**) correspond to specimens evaluated from all 5 patients, and included a minimum of 5 images per specimen. Graphs show median with 95% CI. Mann Whitney test was used to determine statistical significance: **** p < 0.0001.

Palladin expression in the desmoplastic stroma of pancreatic cancer patients correlates with unfavorable prognosis^15^. Further, PDAC associated desmoplasia includes exacerbated ECM deposition of highly aligned collagen fibers that are also associated with poor patient outcome^2^. As an initial approach to establish a link between PDAC-associated ECM alterations and expression of palladin isoforms 3 and 4 (iso3 and iso4), we used multi-photon microscopy to acquire second harmonic generation (SHG) of polarized light, and evaluated the topographic characteristics of bundled collagen in three matched PDAC and tumor adjacent human surgical specimens. The characteristic stromal expansion was evident in PDAC regions (respectively, cyan and magenta regions in Fig. 1a). These expansions included a substantial amount of parallelly oriented collagen fibers (white; Fig. 1a). Not surprisingly, comparing topographical features in fibers detected in PDAC vs. benign tumor adjacent areas, revealed a significant increase (p < 0.0001) in straightened collagen fibers in the tumor (i.e., PDAC) areas (Fig. 1b).

While palladin is known to be upregulated in PDAC stroma^15^, in vivo evidence of palladin correlation with alterations of ECM topography, as well as distinct expression of both isoform 3 (iso3) and isoform 4 (iso4) in PDAC desmoplastic stroma has yet to be confirmed. Thus, the expression of iso3 and iso4 was assessed in the three matched surgical tissue samples used for SHG assessments, by combining the collagen signatures with our established simultaneous multiplex immunofluorescent (SMI) approach^22^, while two additional PDAC and tumor adjacent human tissue pairs were included to improve the rigor associated with these SMI-generated results (Fig. 1a,c). Results, indicated 4- and 2.5-fold stromal increase in iso3 (green) and iso4 (red), respectively, comparing PDAC vs. tumor adjacent tissues (p < 0.0001). These results support previous findings suggesting that palladin is upregulated in PDAC stromal cells^19,23^, and clarify the upregulation of both palladin isoforms (e.g., iso3 and iso4) in the tumor positive stroma that includes substantial production of parallel and straightened ECM fibers.

### Inhibition of TGFβ1 signaling reduces iso3 and iso4 expression as well as CAF-generated ECM alignment

**Figure 2:**
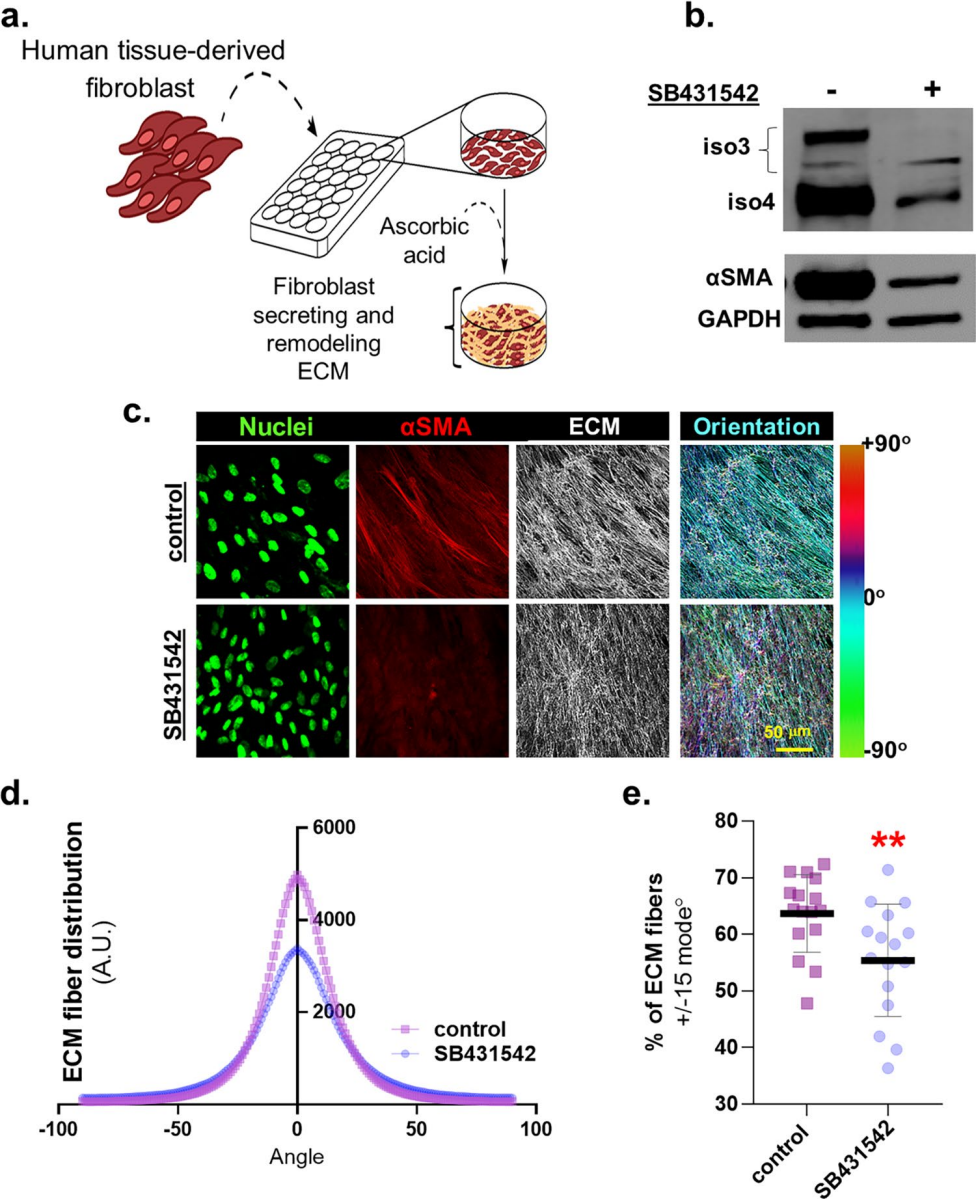
Inhibition of TGFβ1 signaling reduces CAF iso3 & iso4 expression and d-ECM alignment. (**a**) Schematic of 3D fibroblastic cell-derived ECM model used to assess assorted CAF function and/or traits. (**b**) Representative Western blot, using lysates collected at the end of ECM production, and blotted for palladin iso3 and iso4 as well as αSMA expression in vehicle vs. SB431542 treated CAFs. GAPDH served as loading control. (**c**) CAF-derived ECMs were treated during ECM production as in (**b**); shown are representative images of reconstructed confocal image stacks of human fibroblasts in 3D cultures. Images show dye-labeled nuclei in green, immunofluorescently-labeled αSMA (in red), and fibronectin (ECM in white). Scale bar: 50 microns. Last column shows pseudo-colored ECM, depicting corresponding fiber angles distribution, referenced to tone/angle bar on the right; with cyan fibers as the mode angle. Note how ECM fiber analysis of CAFs cultured in the presence of SB431542, included additional fiber color tones suggestive of broader angle distribution compared to control condition. ECM analyses of images acquired in (**c**) were conducted measuring: ECM fiber angle distributions (**d**) and percentage of ECM fibers out of total fibers at 15° from the mode angle (**e**). Data presented were derived from 6 biological replicates (n = 6) and a minimum of 5 images acquired per sample. The Mann Whitney test was used to determine statistical significance. **p = 0.0096.

To query whether changes in expression levels of palladin isoforms occur in response to TGFβ1 inhibition in CAFs in vitro*,* we used our established human desmoplastic 3D culturing system^24,25^ (Fig. 2a). Patient derived CAFs^13,22^ were treated with vehicle control or TGFβ1 signaling inhibitor (SB431542) during d-ECM production. Protein expression was analyzed in cell lysates while protein localization and d-ECM topographies were phenotypically characterized via indirect immunofluorescence. Results show that inhibition of TGFβ1 signaling in CAFs reduced the expression of iso3 and iso4 as well as of αSMA, a biomarker typically used to identify myofibroblastic CAFs^26^ (Fig. 2b). Further, SB431542 treatment did not compromise CAF fibrillogenesis; gauged via ECM thickness (Supplemental Fig. 1a). As expected^22^, CAFs treated with SB431542 produced misaligned ECMs, displayed reduced αSMA expression (Fig. 2c) and ECM fiber distribution was broader in comparison to vehicle treated control CAFs (Fig. 2d). Analysis revealed that SB431542 treated CAFs generated matrices that included less than 60% of fibers distributed at 15 degrees from the measured mode distribution angle (p = 0.0096; Fig. 2e). Taken together, these results confirmed that inhibition of TGFβ1 signaling alters myofibroblastic CAF features (i.e., ECM alignment and αSMA expression) and included limited expression of iso3 and iso4.

### Palladin isoforms are required to sustain myofibroblastic features

**Figure 3:**
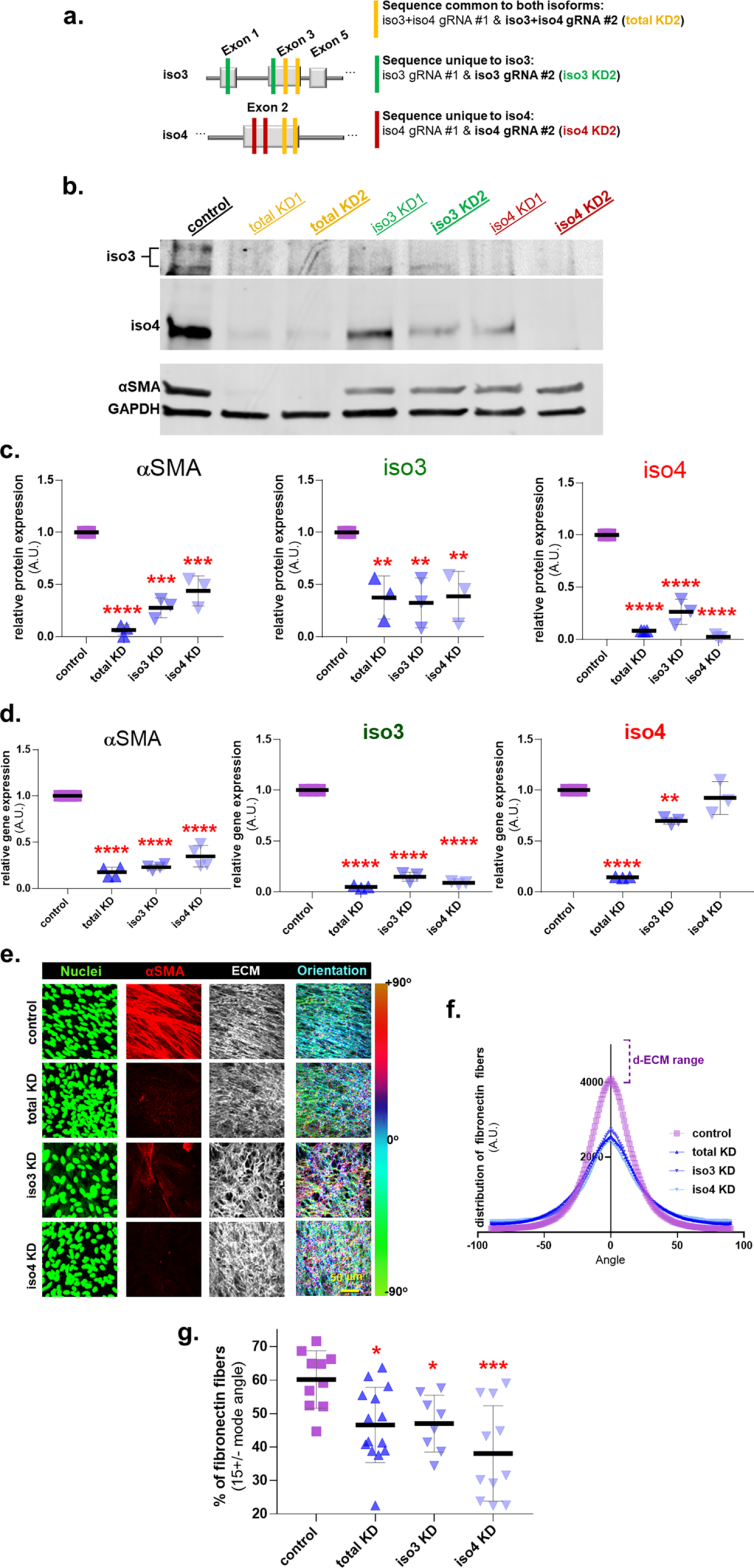
Palladin isoforms are required for CAFs to sustain a myofibroblastic cell identity. (**a**) Schematic illustrating the gRNA strategy (for CRISPR/Cas9) used to generate plasmids that target coding sequence common for both isoforms (total KD: yellow lines) or either palladin isoform (iso3 KD: green line, iso4 KD: red line). (**b**) Representative images of western blot analysis of palladin isoforms and αSMA, using GAPDH as loading controls, in CAFs at the end of ECM production. The upper panel corresponds to the upper portion of the middle panel shown at a higher exposure to highlight iso3 bands, while the middle panel clearly shows iso4. The bottom panel corresponds to αSMA and GAPDH (note that these images were cropped from images generated using the LICOR system). (**c**) Graphs corresponding to measured protein expression levels of samples as in (**b**) normalized to 1 arbitrary unit assigned for expression of the assorted protein/GAPDH in control CAF cultures. (**d**) Graphs depicting relative RT-qPCR quantification of transcripts corresponding to αSMA, iso3, and iso4 normalized to control CAFs, which were set to one arbitrary unit in each experiment. (**e**) Representative images of control vs palladin KD CAFs at the end of ECM production (selecting for substantial ECM produsing samples). Unextracted 3D cultures were subjected to indirect immunofluorescence. Images show in green, nuclei; in red, αSMA and in white, fibronectin (ECM). Pseudo-colored images depict fibronectin fiber angle distributions corresponding to tone bar on the right, showing cyan fibers as mode angle. ECM fibers of control CAFs present greater levels of cyan pseudo-colored fibers compared to palladin KD CAFs. Scale bar: 50 microns. Analyses of images acquired as in (**e**) were conducted measuring: fibronectin ECM fiber angle distributions (**f**) and percentage of ECM fibers out of total fibers at 15° from the mode angle (**g**). Data presented is derived from experimental duplicates of biological triplicates which rendered enough ECM to conduct the meassurements (n = 6). Each sample was represented by a minimum of 5 images. One Way ANOVA, Dunnett’s multiple comparison test was performed to determine statistical significance compared to control CAFs or alignment threshold * p < 0.05, ** p < 0.01, *** p < 0.001, **** p < 0.0001.

In cancer associated desmoplasia, myofibroblastic activation must be sustained to enable CAF-mediated ECM production/remodeling^4^. While palladin is a feature of CAF identity^27,28^, whether both palladin isoforms are required to sustain activation and desmoplastic ECM remodeling remains unknown. To assess the role of iso3 and iso4 in maintaining myofibroblastic CAF activation and a topographically aligned d-ECM, CRISPR/Cas9 was used to generate CAFs deficient in both palladin isoforms and in each isoform individually. To achieve this, two guide RNAs (gRNAs) were designed to simultaneously target both palladin isoforms (total KD1 & total KD2-yellow bars), or each isoform individually; iso3 KD1 & iso3 KD2 (green bars) and iso4 KD1 & iso4 KD2 (red bars) (Fig. 3a). We continued all experimentation with the KD2 cells (referred to as total KD, iso3 KD, iso4 KD CAFs) as these presented with the most efficient downregulation of the intended palladin isoforms (Fig. 3b,c). Figure 3c shows that αSMA protein levels were reduced in all CAF palladin knockdowns (KDs), compared to control CAFs, with the most significant protein loss detected in total KD CAFs (total KD 94%; iso3 KD 72%; iso4 KD 56%). Notably, specifically targeting either palladin isoform reduced the protein expression levels of the other. For example, iso3 was downregulated by ~ 60% in all three KDs, and levels of iso4 slightly varied between ~ 70% reduction in iso3 KD to almost undetectable levels in the other two KD CAFs (Fig. 3c). These data suggest a possible interdependence, regarding the expression of the two isoforms, for the regulation of myofibroblastic CAF features (i.e., high αSMA).

To assess potential epistasis, RNA was isolated from the KD and control CAFs, following sufficient ECM production. RT-qPCR revealed an 80–65% reduction in αSMA and ~ 90% in iso3 gene expression in KD CAFs compared to CAF control (p < 0.0001), while iso4 gene expression was reduced by ~ 85% in total KD (p < 0.0001) and only ~ 30% in iso3 KD CAFs (p = 0.0056) relative to control CAFs (Fig. 3d). Of note, the apparent lack in downregulation of iso4 transcript in iso4 KD, is a result of the designed strategy to introduce a frameshift mutation (see “Materials and methods”). Further, targeting any of the two palladin isoforms, especially simultaneously targeting both (i.e., total KD), suffices to significantly diminish αSMA transcript and protein expressions.

To evaluate whether reducing palladin expression also limits ECM fiber alignment, we queried the fibronectin fiber distribution in the palladin KD CAFs (Fig. 3e–g). While palladin KD often seemed to compromise levels of ECM production (Supplemental Fig. 1b,c), assessment of ECM alignment, on samples that included sufficient ECM production, clearly showed that palladin isoforms are required to maintain a parallel orientation amid CAF-generated ECM fibers (Fig. 3e–g). Thus, limiting palladin expression either results in compromised ECM fibrillogenesis or in loss of d-ECM organization, partially simulating inhibition of TGFβ1 signaling. Collectively, these results suggest that both palladin isoforms could regulate each other and that these contribute to maintaining myofibroblastic CAF features, such as increased αSMA as well as expression and aligned ECM production.

### Palladin isoforms are required to sustain inflammatory CAF features

**Figure 4:**
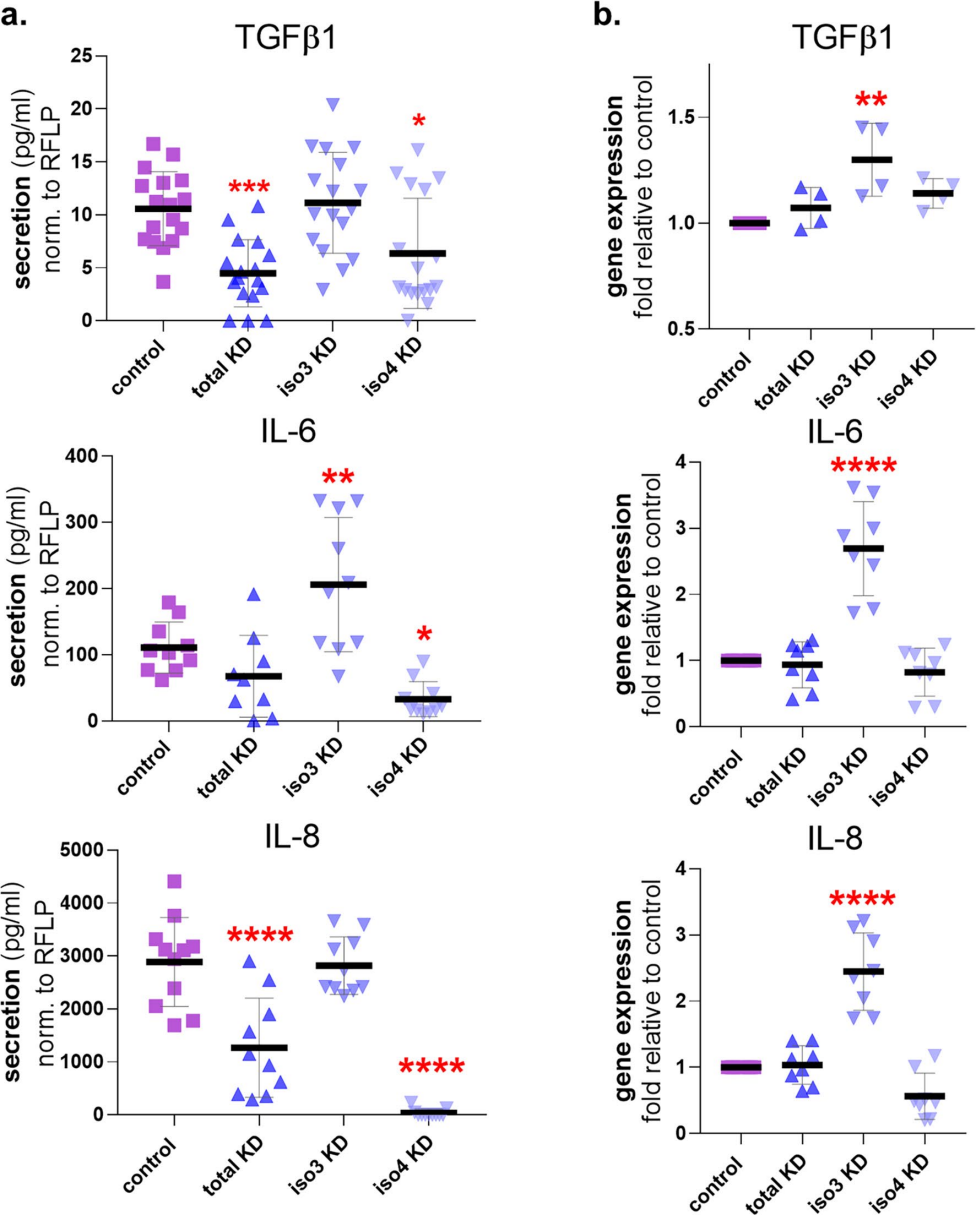
Palladin iso4 is required for CAFs to sustain inflammatory features. (**a**) Measurement of secreted cytokines TGFβ1, IL-6, and IL-8 by palladin KD CAFs normalized to cell number via RFLP expression and compared to control CAF secretions using assorted CAF conditioned media at the end of 5 days ECM production (see “Materials and methods” for additional details). (**b**) Gene expression RT-qPCR transcript analysis of TGFB1 (TGFβ1), IL6 (IL-6), and CXCL8 (IL-8) in palladin KD CAFs, compared to control CAF at the end of 5 days d-ECM production. Data presented were derived from a minimum of 4 experimental replicates of biological duplicates (n = 8). One Way ANOVA, Dunnett’s multiple comparison test was performed to determine statistical significance compared to control CAFs. *p < 0.05; ** p < 0.01; ***p < 0.001; **** p < 0.0001.

One way in which PDAC CAFs participate in tumorigenesis is via secretion of inflammatory cytokines^4,13^. To assess if loss of palladin affects the ability of PDAC CAFs to secrete these and other known CAF-relevant cytokines, conditioned media collected from palladin KD CAFs were analyzed via enzyme-linked immunosorbent assay (ELISA) in which the concentrations of secreted TGFβ1, IL-6, and IL-8 were calculated. TGFβ1 secretion was diminished ~ twofold in total KD and iso4 KD, yet no difference was evident in iso3 KD compared to control CAFs (Fig. 4a). Interestingly, IL-6 secretion was increased almost twofold, in iso3 KD CAFs, and decreased (~ twofold; p = 0.0264) in iso4 KD CAFs while total KD presented no significant differences compared to CAF controls. Further, significant downregulation in IL-8 secretion was evident in total KD and iso4 KD CAFs. To question whether these differences in cytokine secretions could be explained by altered cytokine transcript levels, RNA was harvested from control and palladin KD CAFs and cytokine gene expression was analyzed via RT-qPCR (Fig. 4b). Results showed that TGFβ1, IL-6, and IL-8 gene expression were increased in iso3 KD CAFs compared to control CAFs, whereas levels remained unchanged in the other two KD CAFs (Fig. 4b). Taken together, these results suggest palladin isoforms may play distinct roles in regulating cytokine transcription and secretion and that palladin iso4 may be required for CAFs to effectively secrete TGFβ1, IL-6, and IL-8.

### Palladin isoforms are required to produce pro-tumor desmoplastic ECM

**Figure 5:**
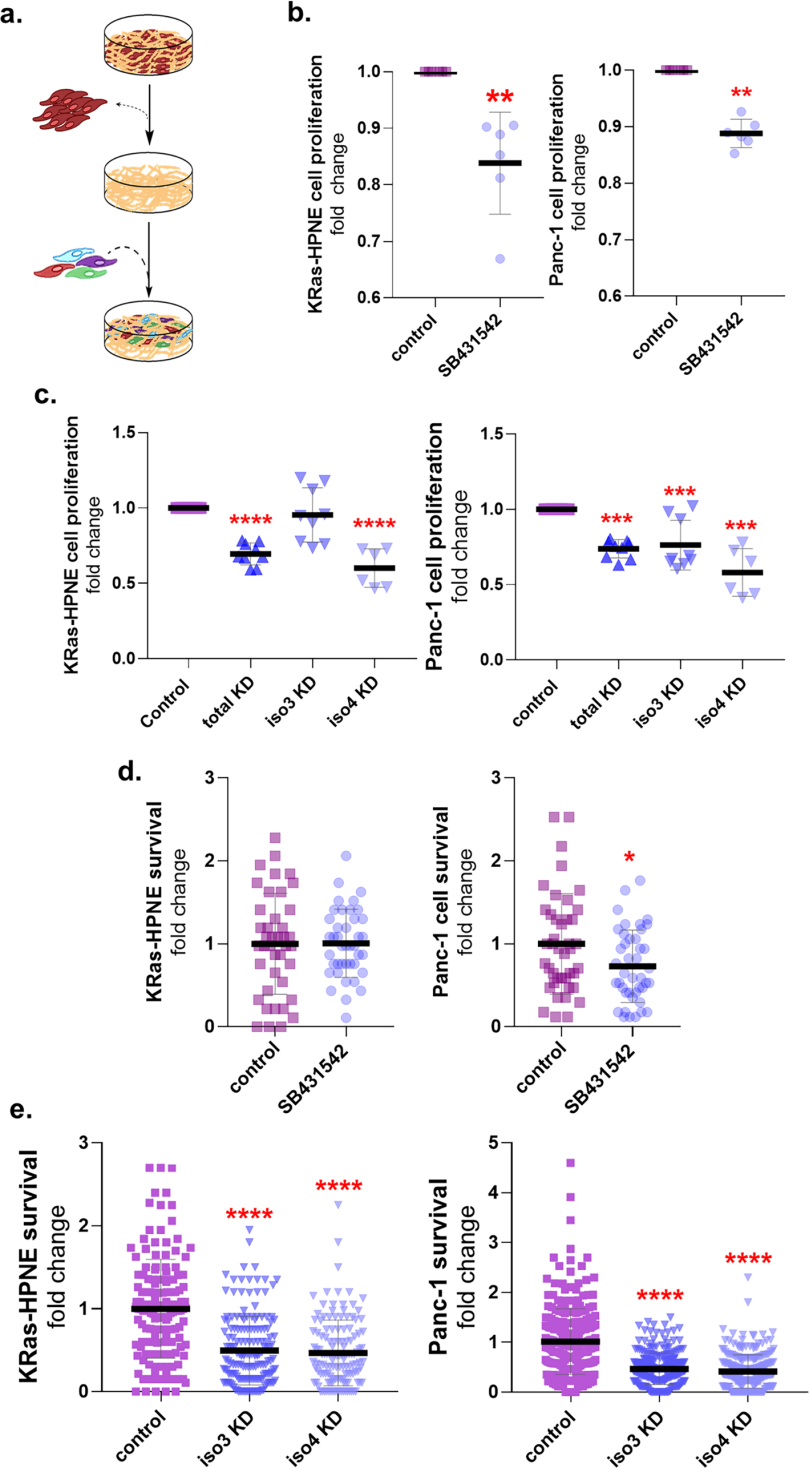
Palladin isoforms are required for CAFs to produce functional, pro-tumor, desmoplastic ECM. (**a**) Schematic of experimental design to assess pro-tumor function of CAF-derived matrices. CAFs are represented by the dark red cells, which are extracted to render cell voided ECMs. Cell free ECMs (deemed substantial following ECM thickness assessments) are re-plated with tumor cells (depicted as multicolored cells) and their proliferation and survival are assessed. (**b**) Graphs depicting metabolic capacity (i.e., proliferation), as determined by the Alamar Blue assay, of KRas-HPNE (LEFT) or Panc-1 (RIGHT) cells cultured in ECMs derived from vehicle or SB431542 treated CAFs. (**c**) Alamar Blue assay conducted to quantify KRas-HPNE and Panc-1 cell metabolic activity (i.e., proliferation) when cells were cultured in ECMs produced by palladin KD CAFs vs control CAFs. (**d**,**e**) RFP expressing KRas-HPNE or Panc-1 cells were cultured in assorted CAF-generated ECMs in the absence of serum and glutamine for 48hrs. Graphs depict the levels of tumorigenic cell survival under nutritional stress compared to Control CAF-generated d-ECMs. Data presented in (**b**,**c**) were derived from experimental triplicates of biological duplicates (n = 6), while data in (**d**,**e**) were derived from experimental triplicates (n = 3) and biological triplicates (n = 9) respectively. A minimum of three images per (**d**,**e**) sample were acquired, dots represent one image. The Mann Whitney Test was performed in (**b**,**d**) while the One-Way ANOVA, Dunnett’s multiple comparison test was performed in (**c**,**e**) *p < 0.05; ** p < 0.01; ***p < 0.001; **** p < 0.0001.

CAFs are known to produce d-ECMs that aid tumorigenesis by supporting PDAC cell growth and survival^4,13,29^. We questioned whether the altered ECM (i.e., sufficient ECM production with reduced ECM alignment), produced by CAFs in experiments described above, could impact the growth of K-Ras driven tumorigenic human pancreatic ductal epithelial cells like KRas-HPNE^30^ and the PDAC cell line Panc-1. Following ECM production, CAFs were extracted from their corresponding ECMs. The cell voided ECMs were used as substrates to culture the tumorigenic cells overnight (Fig. 5a), and cell proliferation was measured via Alamar Blue assay. Control CAFs, treated with SB431542, generated ECMs that led to ~ 20% decrease in KRas-HPNE and ~ 10% in Panc-1 cell proliferation compared to vehicle treated control CAF d-ECMs (Fig. 5b). These findings suggested that during CAF production of d-ECM, TGFβ1 signaling participates in this pro-tumoral myofibroblastic CAF function (i.e., production of d-ECM with pancreatic cancer cell proliferation supportive function). Similarly, we questioned whether ECMs generated by palladin KD CAFs can functionally mimic the effects of limiting TGFβ1 signaling during d-ECM production. Indeed, KRas-HPNE and Panc-1 cell proliferation was reduced overnight in ECMs produced by most palladin KD CAFs as compared to control d-ECMs (Fig. 5c).

We also tested the ability of the various CAF-generated ECMs to rescue KRas-HPNE and Panc-1 cells from nutritional stress-induced death. For this, tumorigenic cell lines were plated in the assorted ECMs for 3 days under serum and glutamine deprivation^13^. Results show that ECMs produced by SB431542 treated CAFs (inhibited TGFβ1 signaling) failed to show differences in supporting KRas-HPNE survival and presented with a modest reduction in Panc-1 cell survival compared to d-ECM controls (Fig. 5d). Notably, the ability of ECMs generated by iso3 and iso4 KD CAFs to sustain CAF generated d-ECM induced tumor cell survival under nutritional stress was impaired by ~ 50% (Fig. 5e). Taken together, these results suggest that the expression of both palladin isoforms is necessary to generate functional d-ECM that supports tumor cell proliferation and survival.

### Palladin deficient CAFs partially respond to TGFβ1 and conditioned media collected from PDAC cells

**Figure 6:**
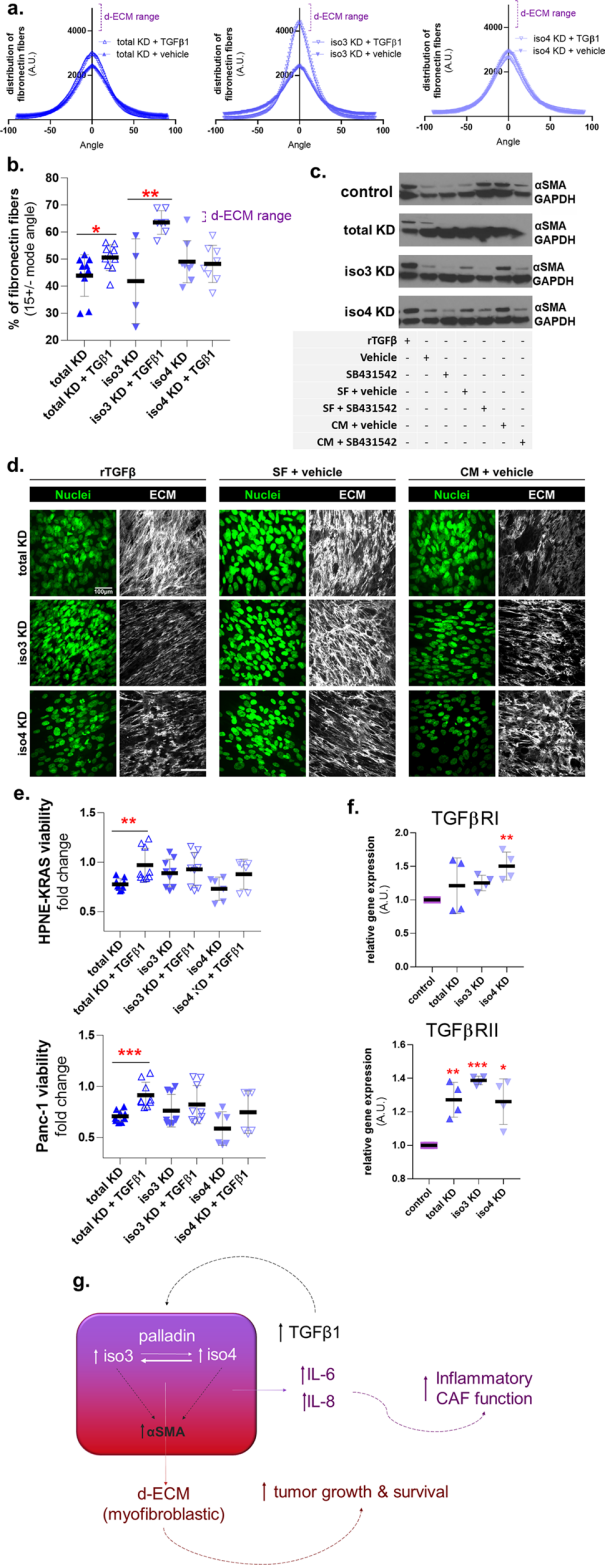
Adding recombinant TGFβ1 to palladin KD CAFs restores some pro-tumor features*.* (**a**) Palladin KD CAFs were treated with vehicle or recombinant TGFβ1 during ECM production. CAF cultures that produced enough ECM were included in these assessments. Analyses of images acquired as before were conducted to measure fibronectin ECM fiber angle distributions and (**b**) percentage of ECM fibers out of total fibers at 15° from the mode angle. (**c**) Palladin KD CAFs were treated with rTGFβ as in (**a**)**,** cultures used represented mutants that did not generate enough ECM and controls were compared to mutant CAFs treated with DMSO (vehicle), TGFβ inhibitor (SB431542), 50% serum free media with complete DMEM plus DMSO (SF + vehicle), 50% Panc1 conditioned media and complete DMEM plus DMSO (CM + vehicle), or 50% Panc1 conditioned media complemented with complete DMEM plus TGFβ inhibitor (SB431542). (**d**) Confocal immunofluorecent images corresponding to (**c**), showing how poor ECM production cannot be restored with rTGFβ or Panc1 conditioned media. (**e**) KRas-HPNE and Panc-1 proliferation in ECMs generated as described in (**a**). (**f**) RT-qPCR transcript analysis of TGFβ1 type I and II receptors gene expression in palladin KD CAFs compared to control CAFs. Data presented were derived from biological triplicates for (**a**,**b**,**e**) three biological replicates in triplicates for (**c**,**d**), and a biological duplicate in (**f**) (n = 4). One Way ANOVA, Dunnett’s multiple comparison test was performed on all analyses where *p < 0.05; ** p < 0.01; ***p < 0.001; **** p < 0.0001. (**g**) Summary cartoon depicting cell autonomous TGFβ1 dependency of CAFs in a palladin dependent manner. The cartoon also shows that while both isoforms regulate each other, and are needed for myofibroblastic features like increased αSMA and production of d-ECM with pro tumoral features, iso4 is mostly needed for inflammatory CAF identity (i.e., secretion of IL-6).

To assess whether stimulating palladin KD CAFs with TGFβ1 ligand would rescue pro-tumor CAF features, we treated palladin KD and control CAFs with recombinant TGFβ1 ligand (rTGFβ1) and assessed restoration of myofibroblastic CAF features, such as production of aligned matrices, as well as ECM-dependent PDAC cell proliferation. Note that as a control, the efficacy of rTGFβ1 treatment was vetted by gauging canonical TGFβ1 signaling via assessment of phosphorylated nuclear SMAD2/3 in CAFs cultured in 2D conditions (Supplemental Fig. 2). In the cases where palladin mutant CAFs generated enough ECM to assess alignment, data shows that rTGFβ1 failed to restore matrix alignment in iso4 KD CAFs, while alignment was modestly increased in total KD and seemingly reestablished in iso3 KD CAFs, achieving levels comparable to alignment ranges observed in d-ECM (Fig. 6a,b). These results suggest that despite the similarities observed amid total KD and iso4 KD CAFs, myofibroblastic features like ECM alignment may not be equally affected in these cells. Nonetheless, in mutant CAFs that did not generate sufficient amount of ECM (~ 50% of the experiments), addition of conditioned media collected from PDAC cells and to some extent addition of rTGFβ, increased αSMA expression (Fig. 6c) but failed to reinstitute sufficient ECM production (Fig. 6d). No differences in cell morphology were evident (not shown).

We next determined whether treating the assorted CAFs with rTGFβ1 renders d-ECMs with a restored ability to sustain tumorigenic cell proliferation overnight, via substantial ECMs voided of CAFs, to assess PDAC cell proliferation (via Alamar Blue assay). Results show that rTGFβ1 partially restored ECM-dependent KRas-HPNE (~ 35%) and Panc-1(~ 20%) cell proliferation only in total KD CAFs (Fig. 6e). We next queried whether TGFβ1 signal transduction could be impeded due to altered TGFβ1 receptor expression in palladin KD CAFs. RT-qPCR showed a tendency for upregulating levels of TGFβR mRNA (Fig. 6f), which may explain why cells were still able to react to rTGFβ1. Together, these data suggest that palladin plays a central role in the production of d-ECMs that sustain tumor cell proliferation while exogenous TGFβ1 induced ECM alignment is dispensable for this role. Overall the data presented suggest an important role for both palladin isoforms in the cell autonomous, TGFβ1-dependent, pro tumoral CAF activation and functions (Fig. 6g).

## Discussion

In addition to its high stromal expression in PDAC^19^, palladin has been reported to be vastly expressed in other neoplastic diseases such as breast, kidney, and colon^27,31–33^. In fact, stromal palladin expression has been clearly associated with patient outcomes in other cancers^15,27^. In effect, a particular palladin mutation, found in “Family X”, who presented with high PDAC penetrance, was shown to strongly complex with alpha-actinin^34^ and to be important for activating myofibroblasts with pro-invasive cancer features^28^. However, in an epidemiological study conducted in 48 patients with familial pancreatic cancer, the Family X mutation was not detected in tumor cells^35^, leading to the speculation that Family X could present a stromal (as opposed to an epithelial) germline pre-disposition. Results from this study agree with the posited stroma-relevant role.

TGFβ1 is an important driver of CAF activation. In fact, we previously demonstrated that inhibition of its canonical signaling reverts pro-tumor CAFs features^22^. However, it is unclear whether TGFβ1-regulated CAF features require palladin iso3 and/or iso4 expression. In this study we explored the contributions of these two major palladin isoforms to known myofibroblastic and inflammatory CAF functions^26^, as well as to CAF-derived pro-tumor ECM-mediated cell proliferation and survival. This study provides evidence to suggest that palladin isoforms play a role in known CAF functions like d-ECM production (able to support tumor growth), and secretion of immunosuppressive cytokines^6,13,26^. Our data suggest that palladin isoform specific roles may vary; especially with regards to TGFβ1-dependent fibroblastic activation^22,36–44^. Experiments using palladin KD CAFs advocate in favor of a palladin isoform interdependency. Although additional experiments will be needed to confirm this idea, previous studies show that iso4 is upregulated during CAF activation prior to induction of iso3 and αSMA expression^19,21^. Since our results showed that iso4 could prevail in the absence of iso3 and, because there was no evidence of iso3 without also detecting iso4, we speculate that iso3 may be regulated by enhanced iso4 levels in CAFs (Fig. 6g).

Notably, significant loss of both isoforms or iso4 alone was necessary for CAFs to limit TGFβ1 secretion. This suggests that the two isoforms participate in a feed forward loop that promotes TGFβ1-dependent desmoplastic field expansion where iso4 may have a more important role^45^. Furthermore, TGFβ1 is known to drive cellular activity via canonical and non-canonical signal transduction pathways that involve Erk1/2 and Akt1/2^46^. Palladin is phosphorylated by both Erk1/2 and Akt1, which was shown to modulate cell migration and contractility^47,48^. Of note, these added aspects of palladin remain to be questioned in PDAC associated desmoplasia and will be interesting to assess in future research investigations.

Regarding known CAF inflammatory cytokines, our data showed that CAFs deficient in iso4 present with limited IL-6 and IL-8 secretion, suggesting these cytokines could be regulated by iso4 alone (Fig. 4). Interestingly, these data are in agreement with the reported pro tumoral function of inflammatory CAFs^13,26^.

Lastly, loss of either isoform resulted in either deficient d-ECM production or in the production of ECMs that were not able to support tumorigenic cell proliferation or survival. This suggests iso3 and iso4 are both needed for the effective production of aligned d-ECMs, known to be pro-metastatic^2^. Yet, similarly to reports showing that loss of NetrinG1 renders aligned CAF generated ECMs that fail to support PDAC survival^13^, results from this study agree with the idea that ECM alignment is not predictive of pro-tumorigenic PDAC proliferation and survival. It would be interesting to examine whether the composition of ECMs produced by loss of either palladin isoform differs from that of control CAFs. This could lead to the identification of pro-tumor ECM proteins to allow distinguishing between ECM organization and ECM composition requirements in sustaining PDAC metastasis vs. survival.

This study provides evidence to support that palladin iso3 and iso4 are increased in PDAC desmoplasia and that downregulation of the isoforms phenocopies many aspects of TGFβ1 signaling inhibition in CAFs, in some ways, even more pronounced than inhibition of TGFβ1 signaling. Results, summarized in Fig. 6g, showed that both palladin isoforms play redundant roles in most CAF functions and that limiting the expression of one prompts the downregulation of the other. Despite some interesting tendencies, results suggest a role for both isoforms in sustain CAFs’ myofibroblastic features. Further, iso4 seems to regulate cytokine secretion (i.e., IL-6 and IL-8), while iso3 appears to be dispensable for this role. Palladin regulation and expression could begin to explain some aspects of the dynamic molecular heterogeneity of CAFs in vivo^3,4,12,26^. Finally, both palladin isoforms are needed for enabling robust canonical TGFβ1 signaling and for CAFs to generate d-ECMs with pro-tumor functions. Thus, further experiments delineating palladin-mediated pro-tumor functions, may aid future therapeutic interventions.

## Materials and methods

### Isolation of fibroblast from human tissue

Human tissues were collected under the approval of Fox Chase Cancer Center’s Institutional Review Board. Patients agreed to donate tissue samples “for research purposes” via signed written informed consent knowing these will be de-identified and results published without any way of knowing who the patients were. Hence, all surgical samples were de-identified, to protect the identity of patients, and distributed for research usage by the Institutional Biosample Repository Facility at Fox Chase. Note that all methods using cells harvested from the surgical samples were conducted in accordance with the guidelines and regulations listed under the journal policies for the “use of experimental animals, and human participants.” https://www.nature.com/srep/journal-policies/editorial-policies#experimental-subjects. Pancreatic tumor surgical samples were received in conical tubes containing Dulbecco’s phosphate-buffered saline (PBS), supplemented with antibiotics, at 4 °C. Samples were minced and subjected to overnight digestion with collagenase at 37 °C^24,25^. Digested tissues were subjected to 10 min of 200 g centrifugation followed by three sequential size-exclusion filtrations through sterile nylon mesh strainers of pore sizes 500 µm, 100 µm and 40 µm. The resulting cells were characterized as CAFs via absence of pan-keratin (AE1/AE3-Dako (Carpinteria, CA)) expression and presence of vimentin (EPR3776-Abcam (Cambridge, MA)) expression according to methods reported previously^24,25^. Characterizations included assessment of ECM alignment, and thickness as published^24,25^. Fibroblasts were then retrovirally transduced as previously described with the pBABE-neo-hTERT vector, (gift from Dr. Robert Weinberg Addgene plasmid # 1774)^22^. To produce a functional retrovirus, Phoenix-Amphotropic (φNX) (ATCC # CRL-3213) packaging cells were grown until 50% confluent in 10 cm culture dishes. Culture dishes were then transfected with pBABE-neo-hTERT vector using Lipofectamine (Invitrogen, #18324) and Plus Reagent (Invitrogen, # 11514015) in 6 mL serum/antibiotic-free Opti-MEM (Gibco, # 31985062) culture media overnight at 37 °C, 5% CO_2_. The next day, cells were refreshed with Opti-MEM (serum/antibiotic-free) and incubated for 24 h at 32 °C. Conditioned medium containing retrovirus was collected and filtered through a 0.45 µm syringe filter (Millipore, #SLHV013SL) on days 2, 3 and 4 post-transfection and immediately used to transduce CAFs.

During transduction, CAFs were cultured in 10 mL of conditioned medium containing the retrovirus supplemented with 4 µg/mL of Polybrene (Santa Cruz, #sc-134220) for 8 h at 37 °C. Then cells were washed with PBS and incubated with fresh culture medium (DMEM 10% FBS, 1% L-Glut, 1% penicillin/streptomycin) overnight at 37 °C. On the following day, this transduction procedure was repeated two additional times, for a total of three retroviral transductions. In the absence of being able to clone these cells, fibroblasts were selected with G-418 until cells reached ~ 90% confluence. Cells were then expanded and tested for overexpression of hTERT and lack of p16 via western blotting to confirm immortalization.

Immortalized CAFs were confirmed to conserve the original ECM production features and used in the study. CAFs were cultured in Dulbecco’s Modified Eagle’s Medium (Mediatech (Manassas, VA)) supplemented with 10% Premium-Select Fetal Bovine Serum (Atlanta Biologicals (Lawrenceville, GA), 2 nM L-glutamine, 100U/ml penicillin, and 100 µg/ml streptomycin (Thermo Fisher Scientific, Waltham, MA), at 37 °C under 5% CO_2_.

### Tumorigenic cell-lines

The tumorigenic cell-lines used in this study to assess cell derived ECM functions were Panc-1 and KRas-HPNE (i.e., E6/E7/Ras/st)^30^. Panc-1 cell cultures were grown in DMEM supplemented with 10% fetal bovine serum (FBS) and 1% penicillin–streptomycin at 37 °C under 5% CO_2._ KRas-HPNE cell cultures were grown in low glucose DMEM media containing 20% M3 Base media (INCELL, San Antonio, TX) supplemented with sodium pyruvate 110 mg/mL, 1% Penicillin–Streptomycin and 5% FBS.

### Cell-derived matrix preparation

Fibroblastic and CAF-derived 3D ECMs were produced using our published protocols^24,25^. Briefly, confluent CAF cultures were maintained for 5–7 days in the presence of freshly prepared ascorbic acid (50 mg/ml), which was added daily. To produce TGFβ1-inhibited CAF-derived ECMs, the following were added during the 5–7 days of ECM production: Vehicle (Dimethyl Sulfoxide) or TGFβ1 Receptor 1 small molecule inhibitor (SB431542; 10 nM final concentration).

### Generating palladin CRISPR/Cas9 KD CAFs

Palladin KD CAFs were generated via CRISPR/Cas9 editing in which specific guide RNAs were designed such that a frameshift mutation was induced in the coding regions common to both palladin isoforms and unique ones were also designed for each isoform (Table 1).

**Table 1:** Guide RNAs.

gRNA	Sequence	Target
TKO1 gRNA FW	**CACCG**TGCTAGAATAGCCTCCGATG	iso3 & iso4
TKO1 gRNA RV	**AAAC**CATCGGAGGCTATTCTAGCA**C**	
TKO2 gRNA FW	**CACCG**GGAACGAAAACTTCGCTTCA	iso3 & iso4
TKO2 gRNA RV	**AAAC**TGAAGCGAAGTTTTCGTTCC**C**	
3KO1 gRNA FW	**CACCG**CAGTAGTGGTTCCATCAGGT	iso3
3KO1 gRNA RV	**AAAC**ACCTGATGGAACCACTACT**C**	
3KO2 gRNA FW	**CACCG**CAAAACACAGCCGTGGCGGA	iso3
3KO2 gRNA RV	**AAAC**TCCGCCACGGCTGTGTTTTG**C**	
4KO1 gRNA FW	**CACCG**TGCGCGGCGATGAACTGCTT	iso4
4KO1 gRNA RV	**AAAC**AAGCAGTTCATCGCCGCGCA**C**	
4KO2 gRNA FW	**CACCG**CGGCGTGCCGTGGCCCGACG	iso4
4KO2 gRNA RV	**AAAC**CGTCGGGCCACGGCACGCCG**C**	

#### Guide RNA (gRNA) design

Guide RNAs (gRNAs) were designed using the MIT Optimized CRISPR Design website: http://crispr.mit.edu/. The first 200 bp of exons 3 and 5 of iso3 coding sequence and exon 2 of iso4 coding sequence (common to both palladin isoforms) and the first 200 bp of exon 1 and 3 of iso3 and exon 2 of iso4 (unique sequences to each isoform) were used to generate gRNAs. The top two hits for each gene were chosen and the gRNA sequences (Table 1) were modified to include BsmBI/Esp3I overhangs (bold) and G (bold, red) to the forward (FW) gRNAs and corresponding C (bold, red) on the reverse (RV) gRNA to optimize its expression under the human U6 promoter.

#### Generation of CRISPR lentiviral vectors

The optimized gRNA sequences were obtained (Integrated DNA Technologies) and cloned into the LentiCRISPR v2 vector (a gift from Dr. Feng Zhang; Addgene plasmid # 52961)^49^. LentiCRISPR v2 is a dual-expression vector that expresses the CRISPR/Cas9 protein along with the cloned gRNA sequence, driven by the human U6 promoter. To generate the complete vector, the gRNA oligos stocks were first diluted to 100 µM and 1 µL of each gRNA pair was added to a T4 PNK reaction mixture (NEB, #M0201S) for every 10 µL reaction. These samples were then placed in a thermal cycler to phosphorylate and anneal the oligos, according to the following program:

- 37 °C for 30 min
- 95 °C for 5 min
- Decrease 5 °C every minute until 25 °C

The annealed and phosphorylated gRNA oligos were diluted 1:200 in RNAase-free water (Thermo Fisher Scientific, #4387937). Meanwhile, 5 µg of LentiCrispr v2 vector was simultaneously cut with Fast Digest Esp3I (Thermo Fisher Scientific, #FD0454) and dephosphorylated with Fast AP (Thermo Fisher Scientific, #EF0651) for 30 min at 37 °C. The cut and dephosphorylated vector was then isolated and purified from a 2% agarose gel using the GeneJet Gel Extraction Kit (Thermo fisher Scientific, #K0691).

The digested and dephosphorylated vector along with the diluted gRNAs were all added to a quick ligation reaction mixture (New England Biolabs, #M2200S) that proceeded for 10 min at room temperature. 2 µL of this reaction mixture was added to 50 µL of competent Stbl3 strain of *Escherichia coli* that was then placed on ice for 30 min prior to heat shocked for 45 s at 42 °C. Immediately following heat shock, samples were placed on ice for 2 min before samples were incubated in S.O.C media at 37 °C for 1 h while shaking. Thereafter, the transformed bacteria were spread onto an LB/agar dish containing 100 µg/mL ampicillin and incubated at 37 °C overnight. The following day, single colonies were screened by colony PCR using the U6 promoter forward primer: GAGGGCCTATTTCCCATGATT and the corresponding reverse gRNA primers (gRNA x.2). Positive clones were selected to expand for plasmid purification and sequencing.

#### CRISPR lentivirus production

Following purification, the cloned LentiCRISPR v2 plasmids and packaging plasmids, psPAX2 (psPAX2 was a gift from Didier Trono; Addgene plasmid # 12260) and VSVg, were added to serum-free/antibiotic-free Opti-MEM along with X-treme Gene 9 transfection reagent (Roche, #06365787001). This transfection mixture was left to incubate at room temperature for 45 min before being added to a culture of 293 T cells containing serum-free/antibiotic-free Opti-MEM media. The following day, the 293 T cell culture was refreshed with DMEM media that was supplemented with 10% FBS and 1% penicillin/streptomycin. Conditioned media was collected and filtered through a 0.45 µM syringe filter (Millipore, #SLHV013SL) on days 2 and 4 post-transfection. Afterwards, the conditioned media containing lentiviruses were immediately used to transduce CAFs or stored at − 80 °C until later use.

#### CAFs transduced with CRISPR lentivirus

CAFs were transduced with CRISPR lentivirus targeting specific palladin isoforms or controls using Polybrene (Santa Cruz, #sc-134220). CAFs were also transduced with a lentivirus overexpressing GFP to which the presence of GFP-positive CAFs signified a successful infection. The following day, media was refreshed on the transduced CAFs and puromycin (2 µg/mL) selection began 3 days post-transduction. Transduced CAFs were selected with puromycin for 14 days and the efficiency of CRISPR/Cas9 targeting palladin was assessed by western blotting. Note that individual CAF clones could not be isolated as these cells become quiescent if cultured as single cells. Hence, the gRNAs that presented with the two greatest loss of palladin protein expression were used for subsequent experiments, which justifies the “KD” terminology utilized throughout the study.

### Human tissue immuno-staining

Simultaneous Immunofluorescence (SMI) was performed on human tumor adjacent or tumor tissue, as done previously^13,22,50^, to access expression levels of palladin isoforms. Briefly, paraffin embedded and formalin fixed (FFPE) surgical patient tissue samples were cut into 5-micron slices and fixed to microscopy glass slides while exposed to a short-wave UV lamp in a light-protected box to quench their natural auto-fluorescence. Sample slides were then stored in a dry, light-protected box until used. Tissue sections were deparaffinized in xylene and rehydrated in ascending graded alcohol to water dilutions. Sections were then treated with Digest-All (Invitrogen) and permeabilized in 0.5% TritonX-100. Samples were blocked using Odyssey Blocking Buffer for 1 h at room temperature prior to incubation with assorted (see below) primary antibodies overnight at 4 °C. Sections were washed using PBS with 0.5% Tween (PBST) and incubated with a mix of mouse monoclonal ‘cocktail’ containing anti-pan-cytokeratin (clones AE1/AE3, DAKO), anti-EpCam (MOC-31EpCam, Novus Biologicals (Littleton, CO)), and anti-CD-70 (113–16 Biolegend (San Diego, CA)^51^ to detect epithelial/tumoral cells, together with rabbit monoclonal anti-vimentin (EPR3776, Abcam) antibodies, indicative of mesenchymal (stromal) components, for 2 h at room temperature. Importantly, antibodies pre-labeled with Q-dots are no longer able to be recognized by secondary antibodies, thus allowing for simultaneous immunofluorescent detection of tumoral and stromal masks together with Q-dot labeled antibodies against “markers”; in this case markers corresponded to palladin iso4 (IE6) and iso3 (G2) tags. Q-dots can be detected when excited in the UV range and using a multi-spectral (liquid crystal filter set/camera) to emit for each specific Q-dot. Sections were incubated with donkey anti-mouse Cy2 (to detect epithelial/tumoral “masked” locations) and donkey anti-rabbit Cy3 (for vimentin positive cell detection), while nuclei were all stained using Draq-5. Prior to acquisition, sections were quickly dehydrated in graded alcohol and clarified in Toluene before mounting in Cytoseal-60. Slides were cured overnight at room temperature before imaging.

### Image acquisitions of FFPE fluorescently labeled sections

Labeled FFPE sections (as depicted above) of patient tissue were imaged using Caliper’s multispectral imaging system (PerkinElmer), which utilizes a unique imaging module with Tunable Liquid Crystal. The Nuance-FX (equipped with a 40 × objective) was used for the multispectral acquisition of “masks” and “markers” described. Negative mock controls were conducted by treating samples to include the specific autofluorescence spectra via non-specific primary antibodies that matched isotypes used for labeling and identical secondary as well as q-dot pre-labeling as above. Once a spectral library was constructed, using each individual fluorophore alone in sequential human pancreas sections, the library was used for subsequent image acquisition and analysis of the samples in question. A high-intensity mercury lamp, equipped with regular filters for FITC, TRITC and CY5 channels, was used to image pre-established compartments masks (epithelial/tumoral = CY2, stromal = CY3 and nuclei = CY5). In combination with corresponding excitation filters, fluorescence emission was collected via long band pass scanning for each excitation wave length as follows: for CY2 (500-720 nm), CY3 (580-720 nm) and for CY5 (680-720 nm). All Q-dot labeled markers were excited using the DAPI filter and respective emissions were collected surveying every 10 λ from 450–720 nm. After collecting all image cubes (including all channels and autofluorescence), images were unmixed to obtain 16-bit greyscale individual monochromatic files for each channel. Using Photoshop’s 'Levels' and 'Batch Conversion' functions, the images were processed in bulk (using pre-set values per channel/marker) to render identically 8-bit scaled monochromatic images for each channel. The resulting images were sampled to set identical threshold values for each channel (to distinguish between signal and noise), which were used to feed the values needed for analyses in our software SMIA-CUKIE 2.1.0 (see below) to signify positive-labeled pixels.

### Simultaneous multichannel immunofluorescence (SMI) analysis

We published this method in Franco-Barraza et al.^22^ and merely re-state it here. SMIA-CUKIE 2.1.0 (SCR_014795) was originally written for the bulk analysis of high-throughput-acquired monochromatic images, corresponding to simultaneously labeled channels used in this and other studies. This software isolates stromal locations while omitting positive tumoral areas, based on mask value thresholds that are provided by the user. Prior to analysis, images (acquired as above) were sorted into ‘Batch Folders’, each containing the five monochromatic images corresponding to the original (unmixed) sample’s epithelium/tumor, stroma, nuclei and palladin iso4 and iso3. The software SMIA-CUKIE 2.1.0, available at https://github.com/cukie/SMIA_CUKIE, was written with the intent of bulk processing and analyzing batches of monochromatic images providing localization (masks), intensities, and quantifying values (markers), including levels and co-localizations of multichannel monochromatic immunofluorescent images. The software can identify intersection areas between an unlimited number of masks, while protein marker values and locations (relative to mask) can also be estimated for numerous interrogations. Finally, paladin iso3 and iso4 stromal values were used for statistical analyses and outputs corresponded to images and graphs provided in Fig. 1.

### Collagen fibers imaging and evaluation

As previously described ^52^, Second Harmonic Generation (SHG) of Polarized Light was collected to evaluate polymerized/fibrillar collagen fibers from human PDAC. FFPE tissue sections were mounted on glass slides and were imaged via SHG^52^. In brief, a water-immersion 25X HC FLUOTAR L 25x/0.95NA W VISIR objective mounted in a Leica SP8 DIVE confocal/multiphoton microscope system (Leica Microsystems, Inc., Mannheim, Germany) was used to image the tissue, which was excited with an 850 nm wavelength generated by multiphoton IR laser Chameleon Vision II (Coherent Inc., Santa Clara, CA). Vimentin positive areas (previously processed as described in Human Tissue Immuno-Staining) were scanned and backward SHG emission from three different areas was captured using a non-descanned detector, set to collect signal between 410–440 nm wavelength. Leica Application Suite X 3.5.5 software was used to acquire selected regions with identical settings and all images were registered as monochromatic, 16-bit image, stacks set to 10 μm Z distances. The stacked images, were tri-dimensionally reconstituted as two-dimensional maximal projections using FIJI (ImageJ 1.52p) software^53^. To study individual collagen fibers’ characteristics, we took advantage of the fiber straightness metric function of the publicly available CT-FIRE software^54^. Collagen fiber straightness evaluation ranged from values between 0–1, in which 1 means a straight fiber and smaller values reflect greater level of fiber waviness.

### SDS-PAGE/western blot

SDS-PAGE followed by western blotting was performed as previously described (Franco-Barraza et al.^22^). Briefly, following matrix production, CAFs were lysed in standard RIPA buffer. Lysates were then homogenized by sonication and allowed to rest on ice before being centrifuged at maximum speed in a microcentrifuge for 15 min. Samples were then diluted in 2 × or 4 × loading buffer (Bio-Rad, Hercules, CA) and boiled for 5 min prior to loading into 4–20% gradient gels (Bio-Rad, Hercules, CA). Gels were run at 100 V for 1.5 h and were subjected to semi-dry transfer to PVDF membranes (Millipore, Billerica, MA). PVDF membranes were blocked for 1 h in 5% milk and 0.1% TBST at room temperature. Membranes were then incubated overnight at 4 °C with the following primary antibodies (Table 2).

**Table 2:** List of SDS-PAGE primary antibodies.

1° antibody	Species	Dilution	Company
αSMA	Mouse	1:10,000	Sigma-Aldrich
Palladin (iso3 and iso 4)	Rabbit	1:5,000	ProteinTech
GAPDH	Mouse	1:10,000	Millipore
pSMAD2/3	Rabbit	1:1,000	Cell Signaling

The following day, membranes were washed in 0.1% TBST, 3 times for 10 min each before incubation with anti-rabbit and/or anti-mouse secondary antibodies (Sigma-Aldrich, St. Louis, MO) in 5% milk in 0.1% TBST. Blots were washed 3 times for 10 min. Protein bands were imaged using an Odyssey Imaging Systems.

### Indirect immunofluorescence (in vitro) and image acquisition

Tri-dimensional fibroblastic culture samples were processed for indirect immunofluorescence as published previously^27,55,56^. Specimens were fixed and permeabilized prior to 1-h blocking using Odyssey Blocking Buffer (LI-COR Biosciences, Lincoln, NE) containing 1% donkey serum. Afterwards, samples were incubated for 1 h with primary antibodies (Table 3) as indicated by the specific experiments.

**Table 3:** List of indirect immunofluorescence primary antibodies.

1° Antibody	Species	Dilution	Company
αSMA	Mouse	1:300	Sigma-Aldrich
Fibronectin	Rabbit	1:200	Sigma-Aldrich
pSMAD2/3	Rabbit	1:200	Cell Signaling

Following three rinses for 5 min with PBS-Tween20 (0.05%), secondary antibodies were incubated for 1 h. SYBR Green (1∶10,000 Invitrogen [Eugene, OR]) or DRAQ5 (1:10,000 Pierce Biotechnology [Rockford, lL]) were used to label nuclei and/or fluorescent Phalloidin (2.5 μl/100 μl, Invitrogen [Eugene, OR]) to label stress fibers were included. Following washes with PBS and a final rinse with double-distilled water, samples were mounted using Prolong Gold anti-fading reagent from Life Technologies (Carlsbad, CA).

Confocal imaging was conducted utilizing a Nikon A1 confocal system, mounted with a plan Apo λ 60X oil immersion objective. To excite the mentioned fluorochromes, the following wavelengths were used: 489 nm for SYBR green or Alexa-488 phalloidin (not shown as no apparent morphological differences were evident); 563 nm for either αSMA or pSMAD2/3; and 638 nm for fibronectin or DRAQ5. Images were acquired utilizing NIS-Elements 5.20 software (Nikon [Melville, NY]). For each acquisition, constant settings were maintained and confocal images were recorded as 16-bit monochromatic stack files. These were processed via FIJI software version 1.52p (Eliceiri/LOCI Group, University of Madison [Madison, WI]) using the “sum” function of z-plane projection obtaining maximal reconstruction of image stacks and preserving their original intensity profile.

### Fibronectin orientation analysis

Samples were indirectly immunostained for fibronectin as described above. Following confocal image acquisition, monochromatic images obtained after maximal reconstruction of fibronectin channel were analyzed using OrientationJ^57^ plugin for FIJI as previously published^22,24^. Values obtained from this analysis were normalized by setting the mode angle to 0° and thereby correcting the angle distribution of all fibers to fall within − 90° and 90° of the mode. The angle distribution for each experimental condition, which consists of a minimum of 5 image acquisitions per condition, were plotted via Microsoft Excel spreadsheets in addition to the standard deviation. The percentage of fibers oriented between − 15° and 15° of the mode was used to determine the ECM alignment of each experimental condition.

### RT-qPCR analysis

RT-qPCR analysis was performed as we have previously done before (Franco-Barraza et al.^22^) Total cellular RNA was extracted from CAFs according to manufacturer’s instructions detailed in the Ambion PureLink kit (Life Technologies). The quality of the RNA was assessed using a Bioanalyzer (Agilent) and RNA concentration was determined with a spectrophotometer (NanoDrop; Thermo Fisher Scientific) following the removal of contaminating genomic DNA via Turbo DNA free from Ambion. RNA was reverse transcribed (RT) using Moloney murine leukemia virus reverse transcriptase (Ambion) and a mixture of anchored oligo-dT and random decamers (IDT). Two reverse-transcription reactions were performed for each experimental duplicate sample using 100 ng and 50 ng of input RNA. Taqman assays were used in combination with Taqman Universal Master Mix and run on a 7900 HT sequence detection system (Applied Biosystems). Cycling conditions were 95 °C, 15 min, followed by 40 (two-step) cycles (95 °C, 15 s; 60 °C, 60 s). Ct (cycle threshold) values were converted to quantities (in arbitrary units) using a standard curve (four points, four-fold dilutions) established with a calibrator sample. Values were normalized to housekeeping gene (RPLPO) and standard deviations were from a minimum of two independent PCRs (Table 4).

**Table 4:** List of RNA probes.

Gene target	Primer sequence
RPLPO (used for normalization including RT-qPCR and ELISA)	FW: CCCATTCTATCATCAACGGGTACAA RV: CAGCAAGTGGGAAGGTGTAATCC
PALLD iso3	FW: CAAGCTGGGAATAAGCGAGAT RV: TCCCTTCACAGAACCATCAGTT
PALLD iso4	FW: AAGGCTCGGAGCCTCCTG RV: ATTTGGTTCTGGAGTTGCTGGA
ACTA2	Hs 00426835_g1
TGFβ1	FW: GAGAGTGCAGAACCGGAGC RV: CATAGATTTCGTTGTGGGTTTCC
TGFβ1 RI	Hs00610320_m1
TGFβ1 RII	Hs00234253_m1
IL-6	Hs00985639_m1
CXCL8 (IL-8)	FW: GCTCTGTGTGAAGGTGCA RV: CCACTCTCAATCACTCTCAGTTC

### Enzyme linked immunosorbent assays (ELISAs): TGFβ1, IL-8, IL-6

Conditioned media (CM) were collected by removing the culture media from Control CAFs (CTL), various KO CAFs (TKO2, 3KO2, 4KO2) following matrix production. CM was used to measure TGFβ1, IL-8, and IL-6 secretion levels via ELISA kits (R&D Systems, Minneapolis, MN) according to manufacturer’s recommendations. Buffers for the TGFβ1 ELISAs were prepared using the DuoSet ELISA Ancillary Reagent Kit 1, while buffers were prepared using the DuoSet ELISA Ancillary Reagent Kit 2 for IL-8, IL-6 (R&D Systems, Minneapolis, MN). Standards and CM collected from KD CAFs were incubated for 2 h on plates pre-coated overnight with capture antibody specific for the cytokine being measured. Prior to measuring TGFβ1 concentration, samples were first incubated with Sample Activation Kit 1 (R&D Systems, Minneapolis, MN) before adding samples to wells. Next, samples were then incubated with detection antibody linked to biotin for 2 h. Afterwards, samples were treated with Streptavidin-HRP for 20 min. Between each incubation, samples were washed 3 times with buffer solutions as recommended by manufacturer. Following Streptavidin-HRP incubation, substrate solution was added to samples for 20 min and then neutralized with stop buffer solution prior to analyzing plates in the Spark Multimode Microplate Reader (Tecan, Switzerland) at 450 nm to measure the specified protein present in sample. Readings were corrected for plate imperfections by also measuring samples at 570 nm and then subtracted from the readings at 450 nm. Samples were also blank adjusted by subtracting the corrected OD readings from media alone from the initial corrected readings for each sample. A standard curve was also generated consisting of a range of known analyte concentrations of the specific cytokines. Corrected readings from samples were then compared to the standard curve to determine the concentration of each cytokine within each sample. The derived concentration was then multiplied by the dilution factor (for IL-6, dilution factor was × 100, for IL-8, dilution factor was × 500). Afterwards, samples were then normalized to RPLPO to account for cell number between each sample.

### Cell proliferation assessment via alamar blue

Cell proliferation was measured using Alamar Blue assay (Bio-Rad) per manufacturer’s recommendation. Briefly, 10% volume/volume alamar blue reagent was added to media, corresponding to CAF generated ECM coated plates (void of CAFs) incubated overnight with tumorigenic pancreatic human cells (i.e., KRas-HPNE and Panc-1). Samples were incubated with the reagent for 4 h at 37 °C. Absorbance was obtained using a plate reader. Readings from wells containing ECMs alone (blank control) were subtracted from readings obtained from experimental wells (i.e., with ECM and cells). The difference was plotted, normalized to controls, and presented in graphs accordingly.

### Survival assay

2 × 10^4^ RFP prelabeled tumorigenic pancreatic cells (KRas-HPNE or Panc-1) were plated onto CAF-voided ECMs generated by control, iso3, and iso4 KD CAFs, in glutamine and serum free DMEM for 48 h. After 48 h, monochromatic 8 bit images (15/well) were acquired on Nikon microscope equipped with epifluorescence and a digital cooled camera, to detect the number of RFP + cells per image. Cell survival/count was quantified using ImageJ. Briefly, a threshold was established to optimize the signal to noise ratio of the RFP signal, and this threshold was applied to every image. Next, a macro that filters out small red particles (< 25 pixels, indicative of debris) and measures % area coverage of the remaining RFP pixels at the previously determined threshold was applied to all images. % area coverage was normalized to control CAF generated d-ECM induced survival, and these values are reported as relative cell survival.

Panc-1 Conditioned Media Treatments on CAFs.

To assess the potential effect of TGF-β signaling induced by PDAC conditioned media (CM) on CAF functions, CON or KD CAFs (1.25 × 10^5^ cells/mL) were allowed to produce ECMs for 5–7 days (as described in “Cell derived matrix preparation”) under the following daily treatment conditions: citric acid, TGF-β ligand (10 ng/mL), DMSO, TGF-β inhibitor SB431542 (10 nM), 50% serum free DMEM and complete DMEM with DMSO, 50% SF DMEM and complete DMEM with SB431542. 50% Panc-1 CM (24 h of conditioning) and complete DMEM with DMSO, 50% Panc-1 CM and complete DMEM with SB431542. After matrix production was completed, CAFs grown on coverslips were subjected to immunofluorescence to detect fibronectin and nuclei, while CAFs grown in 6 well plates were lysed in RIPA buffer and subjected to SDS-PAGE/western blotting to assess αSMA protein expression (see previous methods sections for additional details).

### Statistical analysis

Data presented herein consists of experimental duplicates for every biological triplicate (unless stated otherwise). All image analyses were conducted on at least 5 images containing no less than three cells per region analyzed. Prism 7.0 Graph Pad software (San Diego, California) was used for all statistical analysis. For comparison between two groups the Mann Whitney T test was performed. Groups were deemed statistically significantly different from one another if the p-value was less than or equal to 0.05. For experiments with 3 or more conditions, Dunnett’s multiple comparisons tests were mostly used, unless otherwise stated, using arbitrary values in which control samples were set as 1. Significance was defined as follows: *p < 0.05; **p < 0.01; ***p < 0.001; **** p < 0.0001.

## Supplementary Information

Supplementary Information 1.

Supplementary Information 2.

## Abbreviations

3D: Three dimensional
αSMA: Alpha-smooth muscle actin
Akt1/2: Protein Kinase B
CAFs: Cancer associated fibroblasts
Cas9: CRISPR associated protein 9
CO_2_: Carbon dioxide
CRISPR: Clustered regularly interspaced short palindromic repeats
d-ECM: Desmoplastic ECM
DMEM: Dulbecco’s modified Eagle’s medium
ECM: Extracellular matrix
Erk1/2: Extracellular signal-regulated protein kinases 1/2
F-actin: Filamentous actin
FBS: Fetal bovine serum
gRNA: Guide ribonucleic acid
Iso3: Palladin isoform 3
Iso4: Palladin isoform 4
KD: Knockdown
PBS: Phosphate-buffered saline
PDAC: Pancreatic ductal adenocarcinoma
pSMAD2/3: Phosphorylated SMAD family members 2 and 3
SMAD: Small (worm phenotype) and mothers against decapentaplegic (fly phenotype)
TGFβ1: Transforming growth factor beta1
