# Supplementary material for: Palladin isoforms 3 and 4 regulate cancer-associated fibroblast pro-tumor functions in pancreatic ductal adenocarcinoma
Source: Sci Rep. 2021 Feb 15;11:3802. doi: 10.1038/s41598-021-82937-3 (PMC7884442; doi:10.1038/s41598-021-82937-3)
Supplement: Supplementary file 1 — Supplementary Information 1. [file 41598_2021_82937_MOESM1_ESM.pdf]

Title: Palladin isoforms 3 and 4 regulate cancer-associated fibroblast pro-tumor functions in pancreatic ductal adenocarcinoma

Short Title: Palladin isoforms regulate pro-tumor CAF functions

Authors: Alexander, J.I[1,2]., Vendramini-Costa, D.B[1]., Francescone, R[1]., Luong, T[1]., Franco-Barraza, J[1]., Shah, N[1]., Gardiner, J.C[1]., Nicolas, E[1]., Raghavan, K.S.[1,2]. and Cukierman, E[1,*].

Author Affiliations:
[1] Cancer Biology and the Marvin & Concetta Greenberg Pancreatic Cancer Institute; Fox Chase Cancer Center, Philadelphia, PA.
[2] Molecular, Cellular Biology and Genetics Program, College of Medicine, Drexel University, Philadelphia, PA.

* Corresponding Author: Edna.Cukierman@FCCC.edu ednacukierman@gmail.com
(ORCID 0000-0002-1452-9576)

Supplemental figures

Supplemental Figure 1

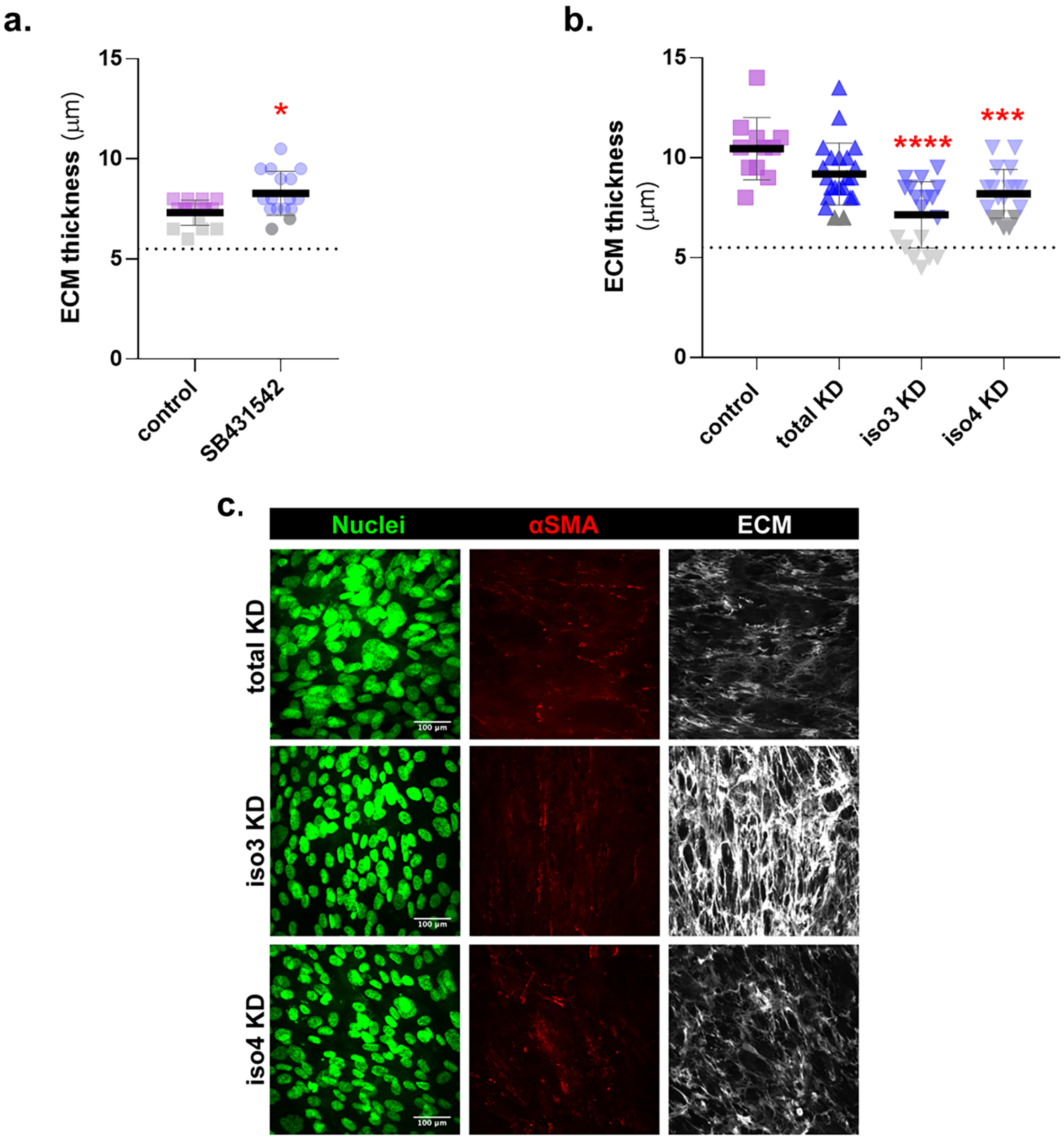

Palladin knock down can compromise ECM fibrillogenesis. Analyses of ECM thickness from samples used to generate graphs presented in main **Figures 2d-e** (**a**) and **3f-g** (**b**). Note that a significant number of matrices obtained from palladin mutants (especially iso3) were compromised in their fibrillogenesis, albeit enough sample presenting with thicknesses measuring ~5.5μm (dotted line) or thicker. To avoid data misinterpretation, matrices thicker than 7 μM (colored as opposed to gray) were used for alignment assessments. The Mann Whitney test was used in (**a**) and one-way ANOVA, Dunnett's multiple comparisons test, in (**b**). Asterisks represent statistical differences compared to control; *$p<0.05$; ** $p<0.01$; ***$p<0.001$; **** $p<0.0001$. **c**. representative confocal indirect immunofluorescent images showing nuclei (green), αSMA (red), and fibronectin (ECM, white), corresponding to the ~50% portion of palladin mutants deficient in substantial matrix production. Scale bars = 100 μm.

Supplemental Figure 2

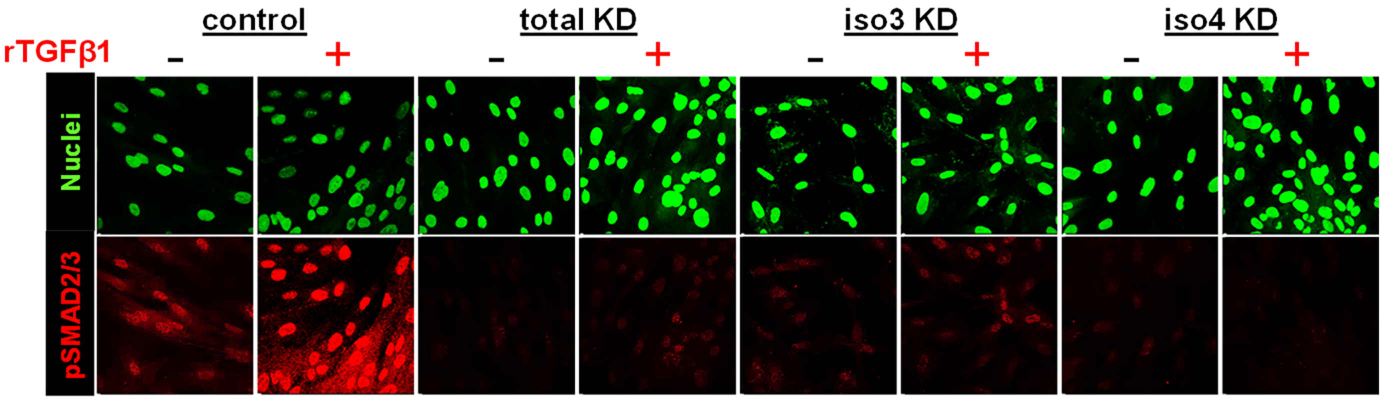

CAFs used in this study can respond to recombinant TGFβ1 treatment. Following overnight serum starvation, control CAFs and palladin KD CAFs, cultured under classic 2D conditions, were treated with recombinant TGFβ1 (rTGFβ1) for 2hrs. Samples were fixed and immuno-stained to observe levels and localization of pSMAD2/3 (red). Results show nuclear (green) localization of pSMAD2/3 was hindered in palladin mutants with the most extreme obstruction evident in iso4 KD CAFs.
