# Supplementary material for: Palladin isoforms 3 and 4 regulate cancer-associated fibroblast pro-tumor functions in pancreatic ductal adenocarcinoma
Source: Sci Rep. 2021 Feb 15;11:3802. doi: 10.1038/s41598-021-82937-3 (PMC7884442; doi:10.1038/s41598-021-82937-3)

Title: Palladin isoforms 3 and 4 regulate cancer-associated fibroblast pro-tumor functions in pancreatic ductal adenocarcinoma

Short Title: Palladin isoforms regulate pro-tumor CAF functions

Authors: Alexander, J.I[1,2]., Vendramini-Costa, D.B[1]., Francescone, R[1]., Luong, T[1]., Franco-Barraza, J[1]., Shah, N[1]., Gardiner, J.C[1]., Nicolas, E[1]., Raghavan, K.S.[1,2]. and Cukierman, E[1,*].

Author Affiliations:
[1] Cancer Biology and the Marvin & Concetta Greenberg Pancreatic Cancer Institute; Fox Chase Cancer Center, Philadelphia, PA.
[2] Molecular, Cellular Biology and Genetics Program, College of Medicine, Drexel University, Philadelphia, PA.

* Corresponding Author: Edna.Cukierman@FCCC.edu ednacukierman@gmail.com (ORCID 0000-0002-1452-9576)

Supplemental Western-blot Images

Original gel (two monochromatic channels) used in main **Figure 2b**

The dotted areas correspond to the cropped portions shown in the main figure. A copy of **Figure 2b** was placed on the top and included dotted areas in yellow and blue corresponding to the areas shown in full disclosed gel in the middle (palladin isoforms) and bottom (alpha-SMA and GAPDH).

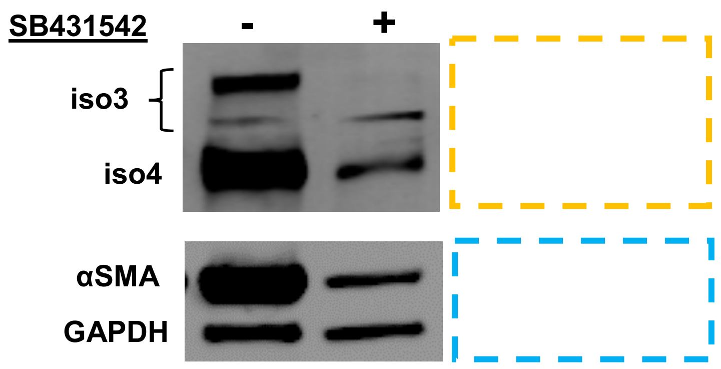

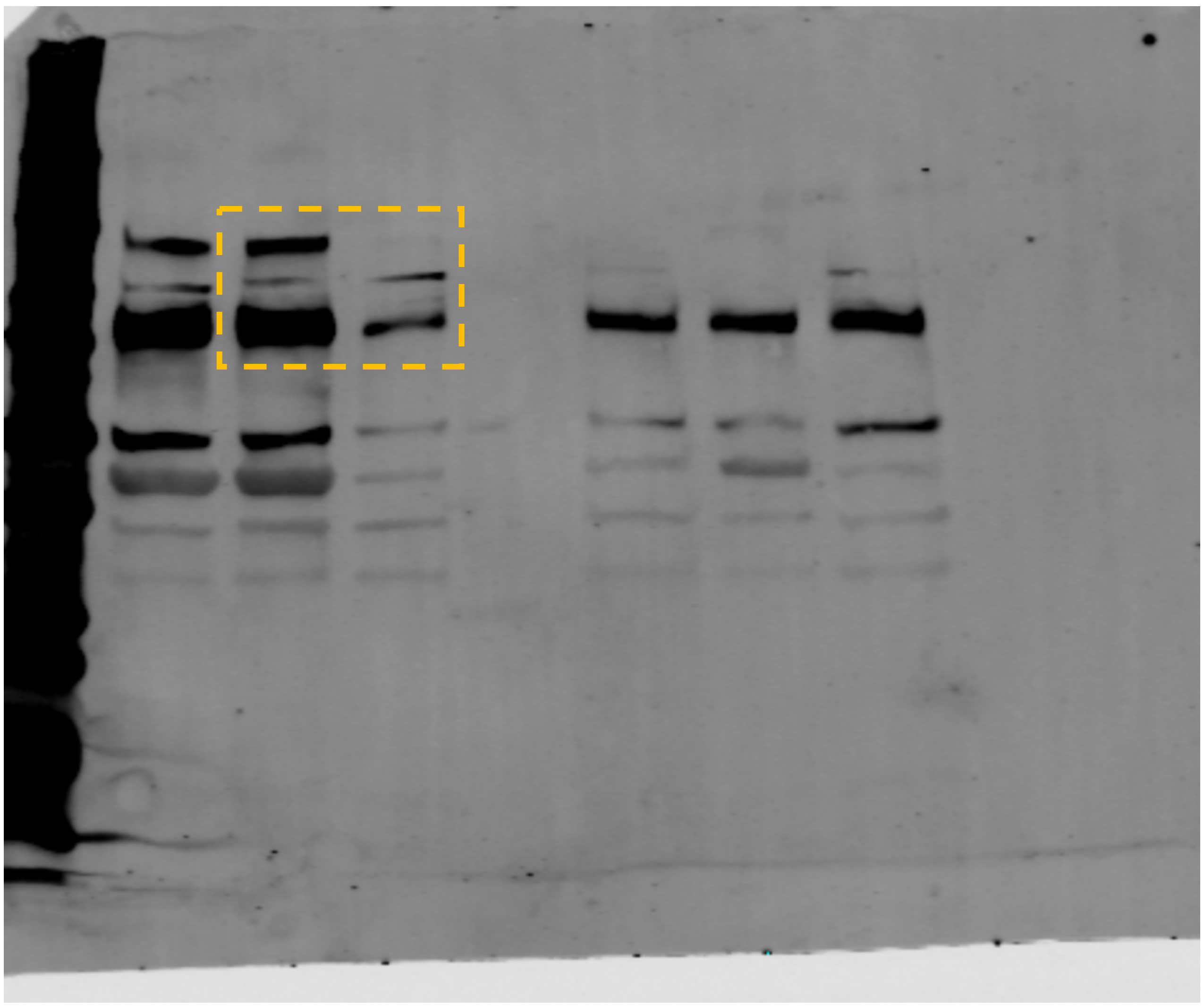

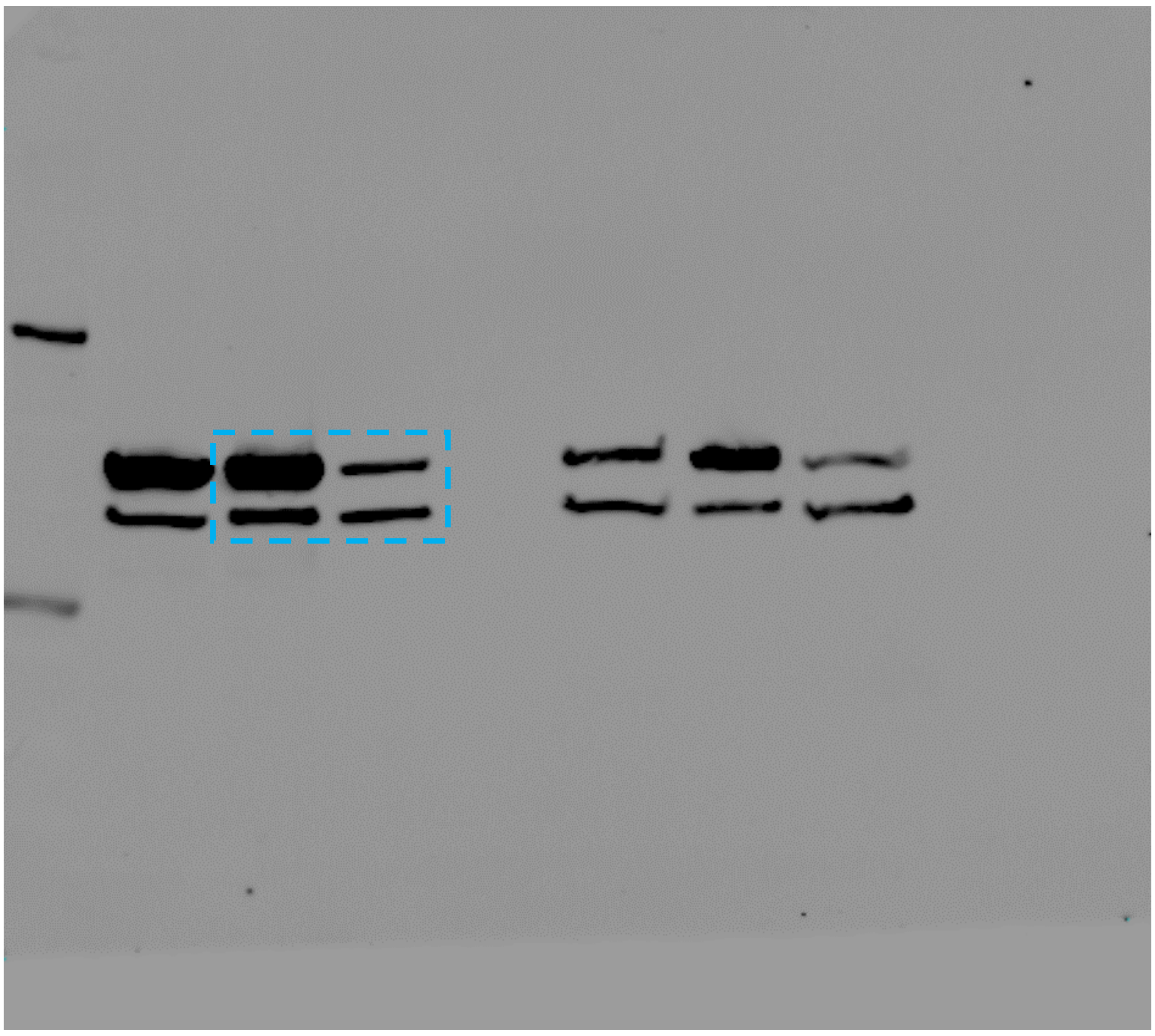

Original gel (two monochromatic images) used in main **Figure 3b**

The upper gel/membrane is the overexposed (increased levels) representation of the membrane shown in the midle panel. This gel (transferred membrane) was incubated against a polyclonal anti-palladin (the isoforms were identified based on their molecular weights). The “overexposed” gel, shown on top, includes the cropped/used portion to the right; yellow dotted area shows exactly how the portion used in **Figure 3b** was cropped. The middle gel/membrane is a lower exposed version of the top and was optimized to show iso 4 changing levels and avoiding saturation (right image shows cropped area used in middle panel of **Figure 3b**). The bottom gel/membrane corresponds to the monochromatic image of the same membrane depicting the monoclonal LiCOR channel and including anti-alpha-SMA (top bands) and GAPDH (bottom bands).

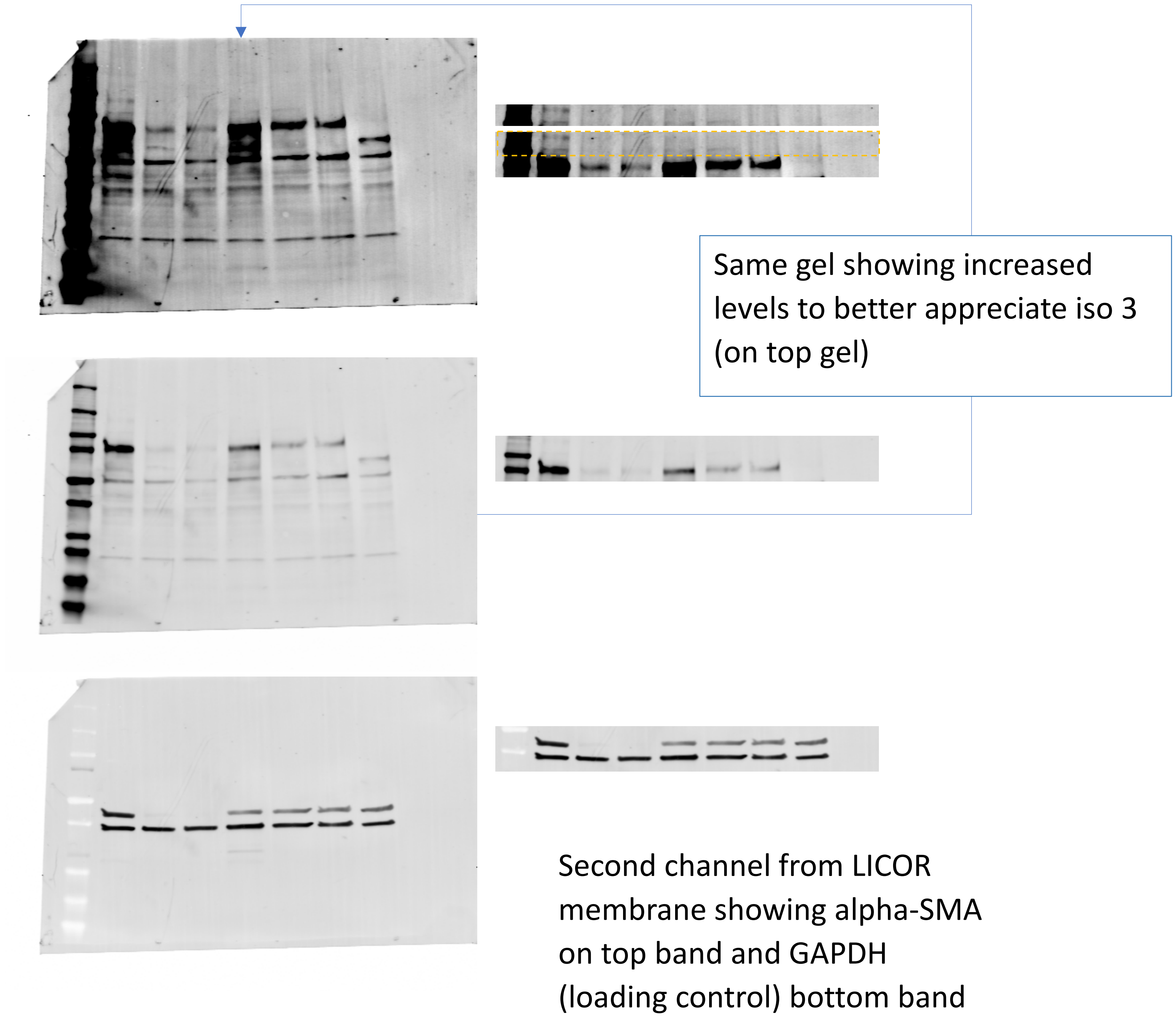

Original scanned films used in main **Figure 6c**

The arrows pointing to the relevant dotted marked areas in full blots (scanned films shown on right including the same three gels at different exposures) correspond to the cropped portions depicted in main **Figure 6c** (left).

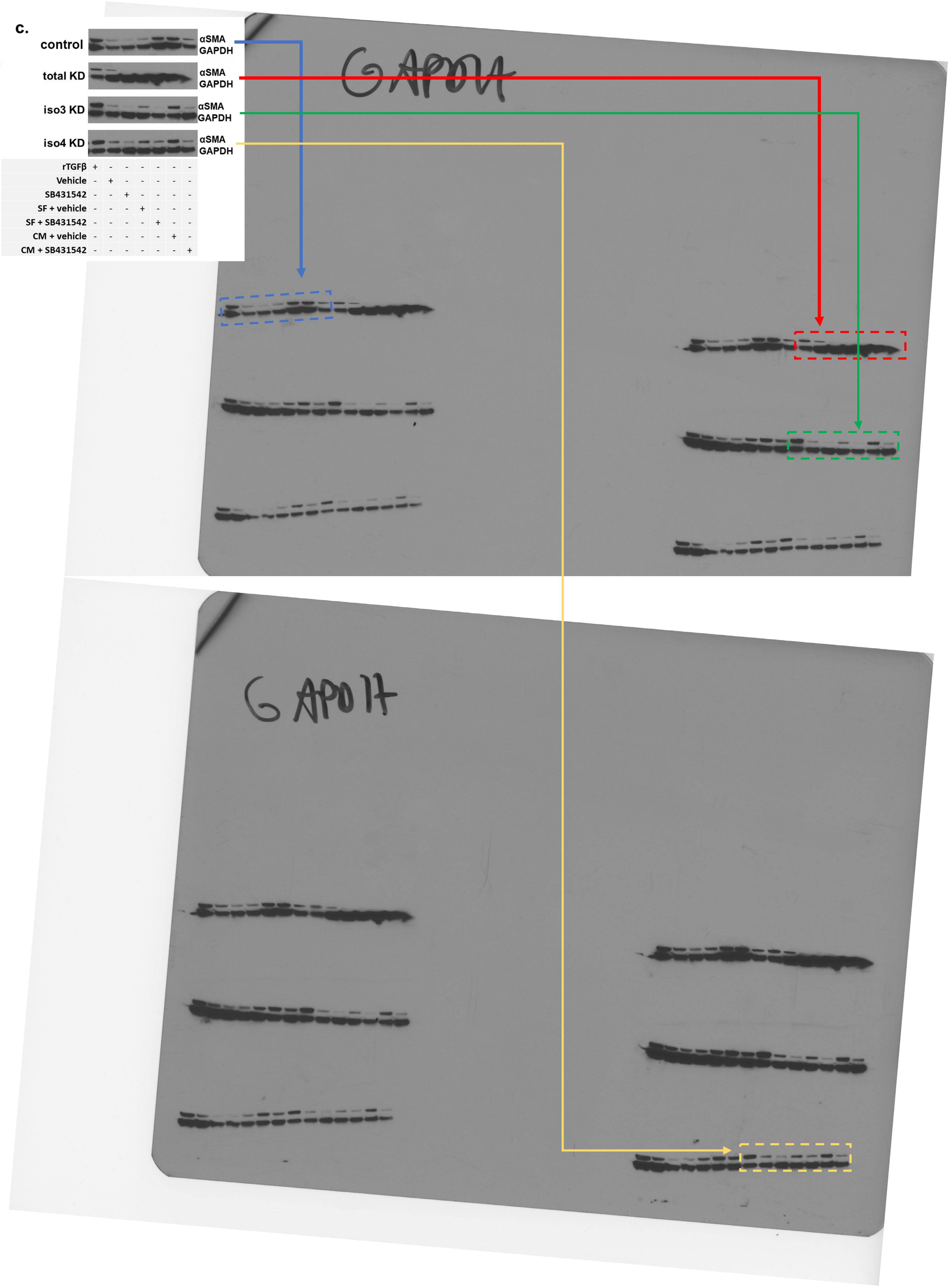

Supplement: Supplementary file 2 — Supplementary Information 2. [file 41598_2021_82937_MOESM2_ESM.pdf]
